# **Repurposing of CNS accumulating drugs Gemfibrozil and Doxylamine for enhanced sensitization of glioblastoma cells through modulation of autophagy**

**DOI:** 10.1038/s41598-025-05054-5

**Published:** 2025-07-01

**Authors:** Smita Dey, Prerika Mathur, Sudeshna Mukherjee, Rajdeep Chowdhury, Syamantak Majumder, Aniruddha Roy, Shibasish Chowdhury

**Affiliations:** 1 Department of Biological Sciences, Birla Institute of Technology and Science, Pilani, Pilani Campus, Vidya Vihar, Pilani, Rajasthan 333031 India; 2 Department of Pharmacy, Birla Institute of Technology and Science, Pilani, Pilani Campus, Vidya Vihar, Pilani, Rajasthan 333031 India

**Keywords:** Glioblastoma multiform, Drug repurposing, Temozolomide, Disease reversal, Autophagy, Drug screening, Cancer, Drug discovery, Oncology

## Abstract

GBM is one of the most aggressive malignancies, having the greatest fatality rate and average life years lost. The current standard medicine, temozolomide (TMZ), is ineffective, requiring the development of new treatments. However, identifying and introducing a novel medicine takes time and money. In this context, repurposing FDA-approved drugs can be a novel yet efficient alternative method. Here, we, therefore, investigated the differential expression signatures of genes of patients suffering from GBM from publicly available GEO datasets and constructed a connectivity map. Functional annotation and KEGG pathway analysis showed dysregulated molecular activities and pathways. Based on their gene ontologies, putative key genes and hub genes linked with the disease were identified, and the C-MAP database was scanned for FDA-approved medicinal compounds that could alter hub gene expression or associated pathways. Our in-silico investigation showed that Gemfibrozil (Gem) and Doxylamine (Doxy) might reverse GBM disease patterns by deregulating GBM-related genes. Evaluation of the GBM inhibitory potential of these drugs through in-vitro and three-dimensional spheroid assay showed promising results. These drugs were more cytotoxic than TMZ; however, they synergised with TMZ as well. Interestingly, the cellular homeostatic process autophagy which has been implicated significantly in GBM pathogenesis and therapy resistance, was found to be inhibited by the drugs Gemfibrozil and Doxylamine, signifying their prospective potential. Therefore, in this study, we, for the first time, identify drugs with the ability to cross the blood brain barrier (BBB), with potential cytotoxic effects beyond TMZ, and with autophagy inhibitory potential, which can be further explored for repurposing against GBM.

**Supplementary Information:**

The online version contains supplementary material available at 10.1038/s41598-025-05054-5.

## Introduction

Glioblastoma multiforme (GBM) is an aggressive malignant brain tumor. It constitutes approximately 12–15% of all intracranial tumors and comprises more than 50% of astrocytic tumors^1^. Even with standard treatment, the median survival post-diagnosis is as low as 10–11 months^2^, with a dismal 5-year survival rate of only 5%^3^ is therefore considered to be highly fatal^4^. Even in the absence of a lower grade precursor, GBM emerges directly into a grade four tumor^5^. Consequently, GBM patients currently undergo a harsh treatment regime, constituting surgical resection and radiotherapy followed by chemotherapy, particularly with temozolomide (TMZ)^6,7^. However, although the current therapeutic procedure improves overall survival percentage, tumors usually recur during or shortly after the treatment course, thus showing resistance to TMZ^8^.

In this regard, several molecular mechanisms have been identified favouring resistance or ineffectiveness of TMZ against GBM^9^, such as expression of DNA repair protein O6‐methylguanine‐methyltransferase, over-activation of DNA repair pathways, over-expression of drug efflux pump and upregulation of survival pathways, like autophagy^10,11^. Also, to address the above challenges, targeted therapies have been explored against GBM. A cancer genome atlas (TCGA) based large-scale genomic and transcriptomic analysis revealed several key pathways, including phosphoinositide 3-kinase (PI3K), receptor tyrosine kinase (RTK), Ras, p53 and retinoblastoma (Rb) signalling pathways to be commonly dysregulated in GBM^9^. However, clinical response against such targeted therapy has been predominantly hindered by intrinsic tumor heterogeneity, ineffectiveness against advanced-stage GBMs, and considerably high costs associated with such therapy, deterring their subsequent use^12,13^. Therefore, discovery of novel targets for drugs that can effectively cross the blood brain barrier (BBB) is warranted for GBM patients. However, an enormous amount of time and huge costs are associated with target identification for drug development and marketing^14^. Also, most of the drugs that show high efficacy in pre-clinical studies fail in clinical trials because of the issue of crossing the BBB^15^. Herein, the time and cost constraints of target-based new drug development can be drastically reduced by using a drug repositioning strategy in which existing FDA-approved drugs are reused for new targets. In recent years, drug repurposing strategies have been adopted in combating many diseases, including cancer^16^, cardiovascular diseases^17^ or neurodegenerative diseases like Alzheimer’s^18^ or Parkinson’s disease^19^. Importantly, a recent detailed review on repurposed drugs in cancer therapy listed a plethora of non-oncogenic drugs that show an encouraging effect on cancer^20^. Herein, because of its ability to cross BBB, repurposed CNS accumulating drugs have primarily shown considerable promise against GBM^21^; however, non-psychiatric drugs like Mebendazole, Vincristine, and Clomifene exhibited encouraging results as well. Notably, most of the existing chemotherapy drugs do not have the potential to eliminate the glioblastoma stem cells (GSC)^22^, which are considered major contributors to the recurrence of malignant glioma and drug resistance^23^. Hence, several recent studies focus on repurposed drugs that target GSCs as well. One such study demonstrated that autophagy modulators positively affect GSCs’ susceptibility to TMZ, which may indicate that modulation of autophagy can be an appropriate strategy^24^. Importantly, drug-induced autophagy is known as an adaptive survival strategy of GBM cells, inducing cytoprotection and consequently facilitating resistance to TMZ. Similar exploration into the identification of newer therapeutic alternatives is required in the future to treat this dreadful disease.

In this study, we employed a drug-centric repurposing strategy to identify existing FDA-approved drugs with potential efficacy against GBM. By integrating gene expression data from GBM patients with the Connectivity Map (CMap) database^25^, we identified two BBB penetrative drugs -Gemfibrozil and Doxylamine capable of reversing GBM-associated aberrant gene signatures. Importantly, our in vitro analysis indicated that these FDA-approved drugs exhibited significantly greater cytotoxicity than the standard drug of choice-TMZ. Furthermore, mechanistic analysis revealed that both compounds can potentially inhibit autophagy, the cellular homeostatic pathway often elevated and implicated in GBM pathogenesis. Moreover, Gemfibrozil and Doxylamine also showed synergistic effects with TMZ. Our study strongly suggests that these repurposed drugs can be explored for further analysis and may serve as viable alternatives to TMZ for the treatment of GBM.

## Results

### Identification of differentially expressed genes in GBM patients and their functional enrichment

**Fig. 1:**
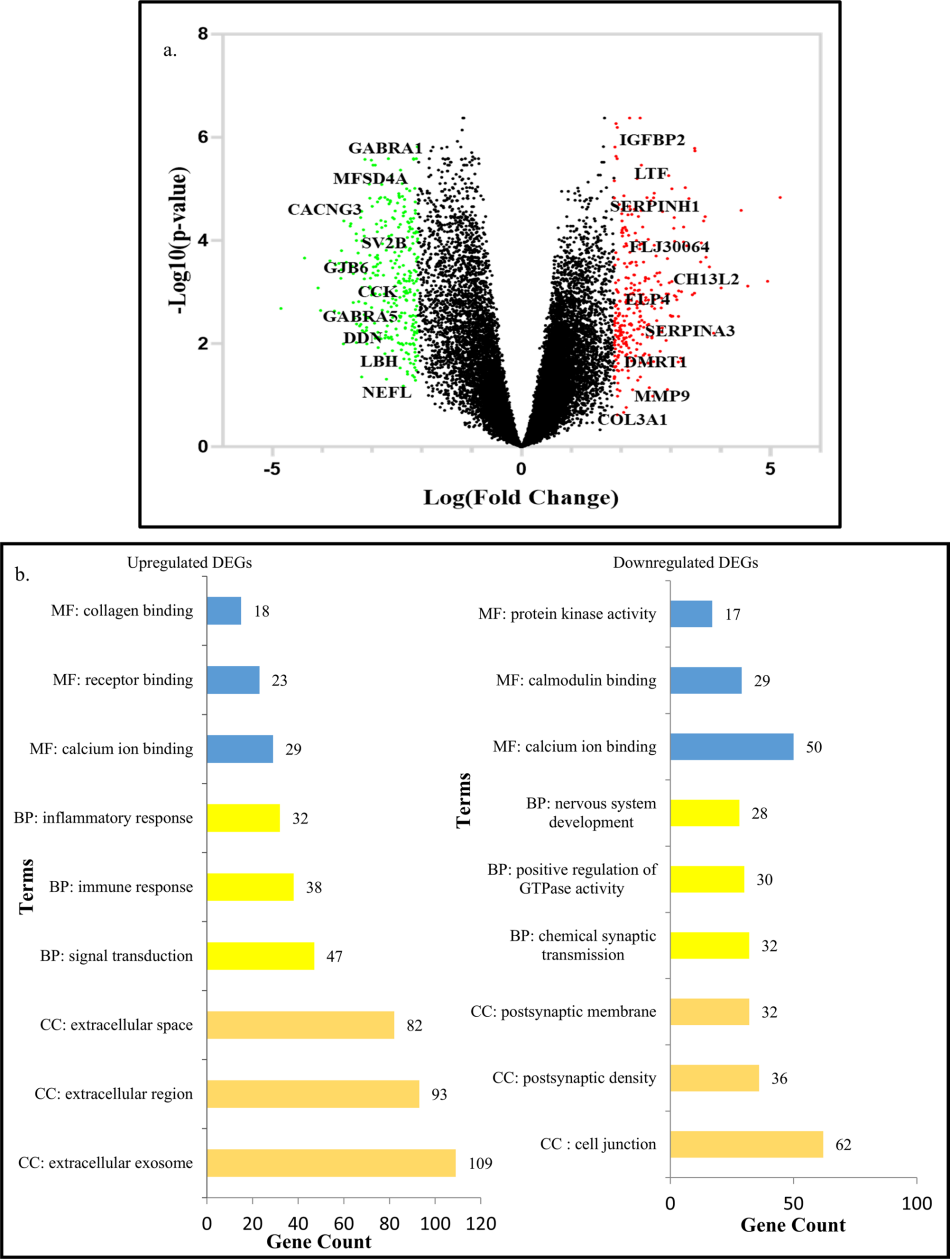

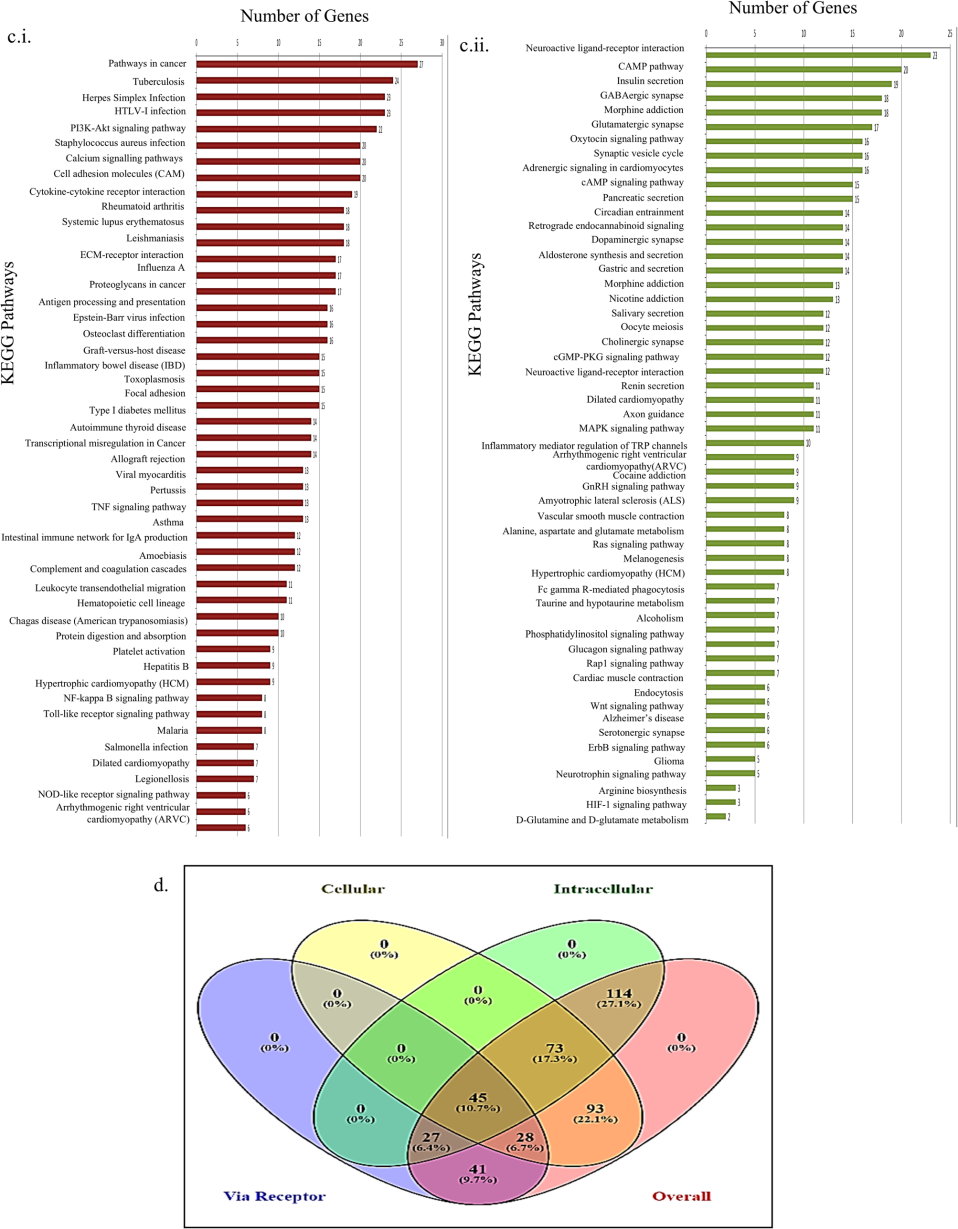
Identification of differentially expressed genes in GBM patients and their functional enrichment: (**a**) Volcano Plot of all the genes obtained on comparing the GBM samples from patients to normal brain samples. All the red dots correspond to the differentially upregulated genes, while all the green dots correspond to differentially downregulated genes. (**b**) Gene Ontology (GO) analysis of differentially expressed genes (DEGs) using DAVID (i) Functional annotation of upregulated DEGs. (ii) Functional annotation of downregulated DEGs (**c.i**) Bar graph representing all the significantly enriched KEGG pathways^81^ associated with upregulated DEGs, (**c.ii**) Bar graph representing all the significantly enriched KEGG pathways^81^ associated with downregulated DEGs, (**d**) Venn Diagram representing differentially expressed genes which were found to regulate cellular processes, intracellular processes, and receptor-mediated processes.

To understand the cohort of genes that are dysregulated in GBM patients, we analysed transcriptomic data from GBM patients (GSE2223 and GSE 4290). We found that among a total of 24,067 dysregulated genes, 20,441 genes were downregulated, while 3,626 genes were upregulated in GBM patients. Using log2 fold change and *p*-value cutoffs, 1,062 significantly differentially expressed genes (DEGs) were identified^26^ in GBM patient samples compared to normal brain tissue. The volcano plot (Fig. 1A) highlights 329 upregulated DEGs (red dots) and 733 downregulated DEGs (green dots). The top 10 upregulated and downregulated genes identified (on the basis of log2 fold change) in this analysis are labeled in the Fig. 1A and listed in Table 1A and B, respectively. These genes may be relevant to understanding GBM pathogenesis and identifying potential therapeutic targets. The biological significance of the significantly differentially expressed transcripts was assessed through gene ontology (GO) analysis, followed by categorization into cellular mechanism, intracellular signalling, and receptor-mediated signalling pathways. Gene Ontology (GO) analysis revealed that most upregulated genes were associated with biological processes such as signal transduction, immune responses, and inflammatory responses. In contrast, downregulated genes were primarily involved in chemical synaptic transmission, positive regulation of GTPase activity, and nervous system development (Fig. 1B).

**Table 1:** The list of top 10 (A) upregulated and (B) downregulated differentially expressed genes (DEGs) along with their p value and log fold values, identified by comparing GBM samples (GSE2223 and GSE4290) to normal controls.

Gene	LogFC	Adj. *p*-value
(A)		
IGFBP2	5.118944	1.11E−20
LTF	5.007242	8.28E−09
SERPINH1	4.580033	9.91E−17
FLJ30064	6.7652	4.21E−22
CHI3L2	6.24685	6.27E−20
ELP4	6.1282	5.89E−23
SERPINA3	6.034	1.03E−10
DMRT1	5.62624	5.50E−20
MMP9	5.27793	3.44E−17
COL3A1	5.03023	8.89E−06
(B)		
GABRA1	− 5.7117	7.68E−11
MFSD4A	− 5.2889	2.41E−14
CACNG3	− 5.2357	7.31E−17
SV2B	− 5.1258	6.94E−12
GJB6	− 5.1185	4.47E−09
CCK	− 5.0016	1.24E−10
GABRA5	− 4.9056	1.35E−14
DDN	− 4.9053	1.14E−14
LBH	− 5.2987	1.30E−14
NEFL	− 4.9786	1.15E−14

The selection was based on their log fold change (logFC) values, where a negative logFC corresponds to the downregulated genes while positive value indicates upregulated genes.

KEGG pathway analysis further showed that upregulated DEGs were enriched in the PI3K-Akt signalling pathway and cytokine-cytokine receptor interactions. In contrast, downregulated DEGs were predominantly associated with insulin secretion, cAMP signalling, and GABAergic synapse pathways (Fig. 1C). Additionally, Venn diagram analysis (Fig. 1D) identified 45 ‘key’ genes common to three functional subcategories: cellular processes, intracellular signalling, and receptor-mediated signalling^27^. These key genes may play a critical role in GBM progression. Among them, the upregulated genes were significantly enriched in pathways such as cytokine-cytokine receptor interaction, IL-17 signalling, and AGE-RAGE signalling in diabetic complications, whereas the downregulated key genes were enriched in the GABAergic synapse and cAMP signaling pathways.

### Protein–protein interaction (PPI) network of the ‘key’ genes and identification of hub genes

**Fig. 2:**
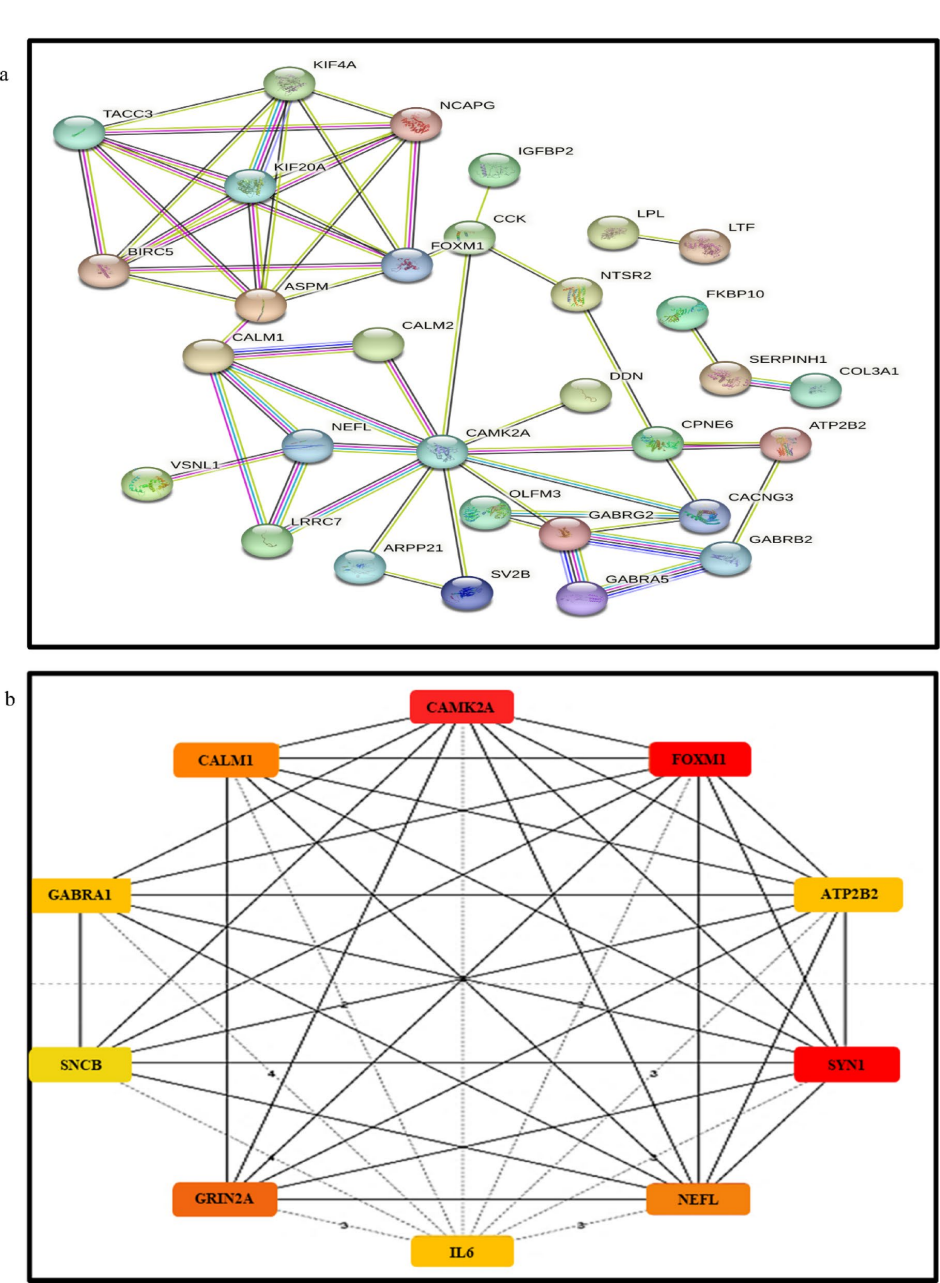
Protein–protein interaction (PPI) network of the ‘key’ genes and identification of hub genes: (**a**) Protein–Protein Interaction network of the 45 key genes generated using STRING, (**b**) A network of ten hub genes were obtained using cytoHubba, an application of Cytoscape. The nodes are colored based on their scores obtained using MCC. The darker the node, the greater its score (red) and the lower scores are represented as yellow.

Cellular life depends on a complex network of functional interactions between biomolecules. Among these, protein–protein interactions (PPIs) are especially significant due to their versatility, specificity, and adaptability. To explore these interactions, we constructed a PPI network of the 45 “key” genes using STRING. A total of 152 PPI relationships with a combined score > 0.4 were identified, with an average node degree of 6.76. The network was then analysed using cytoHubba, which identified the top ten hub genes based on the maximal clique centrality (MCC) method. These hub genes, CAMK2A, FOXM1, SYN1, CALM1, GRIN2A, NEFL, GABRA1, IL6, ATP2B2, and SNCB were found to be highly interconnected (Fig. 2A). The MCC method assigns scores to nodes, with darker nodes indicating higher scores, as detailed in Table 2. Further analysis revealed that these hub genes play key roles in regulating dysregulated pathways in GBM, such as calcium signalling and insulin secretion (Fig. 2B).

**Table 2:** List of top ten key genes are listed along with MCC score is shown. Based on their scores, the hub genes were identified.

Rank	Name	Score
1	CAMK2A	1721
2	FOXM1	1659
3	SYN1	1422
4	CALM1	1336
5	GRIN2A	1128
6	NEFL	915
7	GABRA1	873
8	IL6	720
8	ATP2B2	696
10	SNCB	620

### Analysis of GBM gene signature reveals drugs that can be repurposed for GBM

**Fig. 3:**
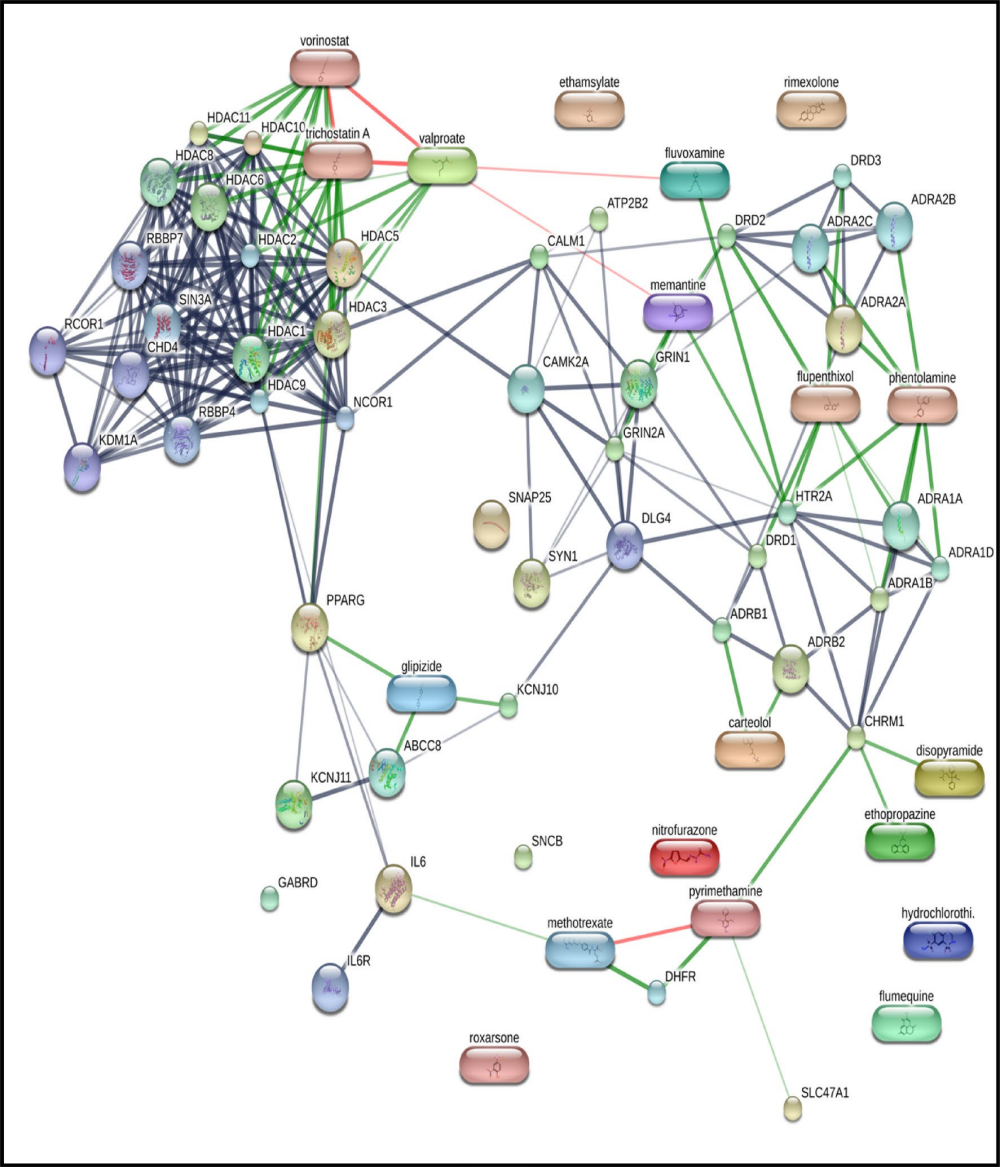
Analysis of GBM gene signature reveals drugs that can be repurposed for GBM: PPI network of hub genes predicted for GBM and the predicted drugs, along with the gene targets of the predicted drugs. This network was generated using STITCH. The drugs are depicted as rectangular boxes, while the proteins are shown as circles.

The top 45 differentially expressed key genes identified from patient data were used as input in the Connectivity Map (CMap) database to identify potential drugs that could reverse the GBM signature. Each query gene, whether upregulated or downregulated, was compared to the CMap reference database, producing a rank-ordered list of signatures based on their connectivity scores. Drugs with high negative connectivity scores were selected and further filtered based on molecular weight, polar surface area, and the logarithm of partition coefficient, ensuring that they could effectively cross the blood–brain barrier and potentially be therapeutically viable for GBM. Additionally, a drug-protein interaction analysis (Fig. 3) was performed using Cytoscape, inputting the 45 key genes and the top 25 drugs to explore their interactions. This analysis showed that several key genes identified in this study are primary targets for drugs previously tested on the U87 cell line^28,29^. Drug perturbation data from the GDSC portal confirmed that these drugs share mechanisms and pathways relevant to GBM treatment. From the top 25 drugs, Valproic Acid (VPA), Gemfibrozil (Gem), Doxylamine (Doxy), and Diprophylline (Dipro) were further selected for their ability to cross the BBB and modulate autophagy^30^. The drug-protein interaction analysis revealed that VPA is targeting GABA receptors and voltage-gated ion channels in addition to inhibiting HDAC. Gem is a lipoprotein lipase inhibitor targeting PPARα, Doxy is an H1-receptor antagonist, and Dipro is an adenosine receptor antagonist. A summary of the shortlisted drugs, their classifications, and the key genes they target is provided in Table 3.

**Table 3:** The list of selected drugs for in-vitro experiments was identified using the Connectivity Map.

CMap name	Cell line	Class of drug	Drug targets	Mol. Wt	TPSA	LogP
Valproic Acid	HL60	HDAC Inhibitor	ACADSB, HDAC 9, OGDH, ALDH5A1	144.21	37.3	2.8
Gemfibrozil	PC3	Lipoprotein Lipase Inhibitor	PPARA, SLCO1B, SLC22A8, SLCO2B1, SLCO1B3	250.33	46.53	3.61
Doxylamine	PC3	Histamine Receptor Antagonist	CHRM1, HRH1	270.36	25.36	2.96
Diprophylline	MCF7	Adenosine Receptor Antagonist	ADORA1, ADORA2A, ADORA1, ADORA2A, PDE3A, PDE4A, PDE4B, PDE4C, PDE4D, PDE7A, PDE7B	254.24	98.9	− 1.9

These drugs were chosen based on their negative connectivity score, ability to cross the blood–brain barrier (BBB), and autophagy-modulating properties. Drug name with their probable target genes along with molecular weight, topological polar surface area (TPSA) and lipophilicity (LogP) value of drug is listed.

### Doxylamine and Gemfibrozil induce cytotoxicity in GBM cells

**Fig. 4:**
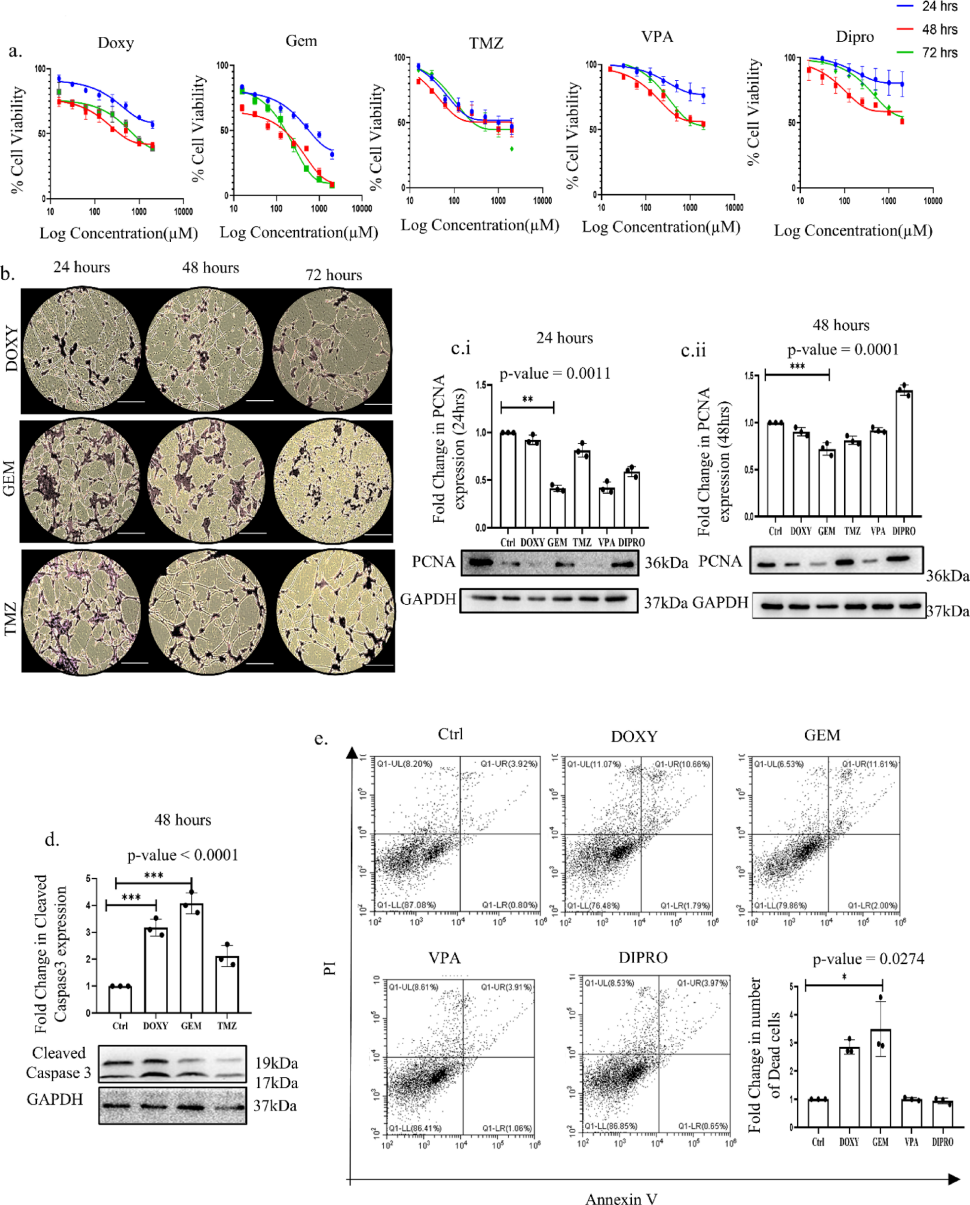
Doxylamine and Gemfibrozil induce cytotoxicity in GBM cells: (**a**) Bar graph representing dose and time kinetics of Doxylamine (Doxy), Gemfibrozil (Gem), Diprophylline (Dipro), and Valproic Acid (VPA), and compared with the Standard Drug TMZ by MTT assay, (**b**) Crystal violet images capturing the viable cells after 24 h, 48 h, and 72 h of drugs treatment, scale bar represents 10 μm (**c**) Immunoblot showing expression of proliferation marker PCNA after (**i**) 24 h and (**ii**) 48 h of incubation, (**d**) Cleaved Caspase 3 expression by Immunoblotting confirming apoptosis of the cells (cropped image of the blot is provided), (**e**) Analysis of the total number of cell death by AnnexinV/PI data assay obtained after 48 h of drug treatment. *p*-value ≤ 0.05 is considered significant, and * refers to a *p*-value less than or equal to 0.03, ** refers to a *p*-value less than or equal to 0.002, and *** signifies a *p*-value less than 0.0001 between control and different test groups. All experiments were performed in triplicate unless stated otherwise, and all data were generated using the U87 cell line, unless specified differently.

To validate the GBM inhibitory potential of the drugs shortlisted through in silico analysis, we thereafter performed wet lab experimentation. Various studies have indicated that the IC_50_ value of TMZ ranges from 4000 to 875 µM^31–33^. Thus, in-vitro cytotoxicity was initially analyzed through MTT assay in established GBM cell lines U87 and U373 to establish the IC_50_ values of the drugs and also compared with that of TMZ. Doxy and Gem were found to have the highest cytotoxic activity in U87 cells (Fig. 4A); importantly, the IC_50_ values of these drugs were found to be considerably lower than the conventionally used drug, TMZ. The IC_50_ value of Doxy was found to be around 600 µM, and that of Gem is 250 µM, while that of TMZ is around 1250 µM, indicating that both Doxy and Gem can be a better alternative to TMZ. However, the two other drugs selected for the study—Dipro and VPA- did not show any significant cytotoxic effect during the time period studied (Supplementary Fig. 1A). In addition, images of the U87 cells captured post-Doxy and Gem treatment corroborated the cytotoxic effect observed earlier (Fig. 4B). Thereafter, to confirm the cytotoxic effect of the drugs, we analyzed the protein expression of proliferating cell nuclear antigen (PCNA), a well-established cell proliferation marker, which showed a considerable decrease after 24 and 48 h of Doxy and Gem treatment (Fig. 4C). Furthermore, an elevated level of cleaved caspase 3 and an increased Annexin V/PI positive U87 cells, an indicator of activation of apoptosis, was observed after Doxy and Gem exposure (Fig. 4D,E). Importantly, studies on U373 cell line also displayed similar responses to the drugs. The U373 cells also showed significant cytotoxicity to Doxy and Gem as analyzed by MTT assay, phase contrast images, and crystal violet staining (Supplementary Fig. 1B–D). In corroboration with results obtained from studies in U87 cells, we observed an increased Annexin V and PI population, reduced PCNA expression, and increased cleaved Caspase 3, confirming the putative effect of the drugs in GBM cell types (Supplementary Fig. 1E–G). Furthermore, these drugs showed enhanced cytotoxicity when exposed in conjunction with the standard treatment for Glioblastoma, TMZ (Supplementary Fig. 1H). Overall, our findings show that both Doxy and Gem are cytotoxic to GBM cell types, have considerably enhanced cytotoxicity than TMZ, and can show potential synergy with the conventionally used drug as well.

### Gemfibrozil and Doxylamine show cytotoxicity in 3-D spheroid cultures of GBM cells

**Fig. 5:**
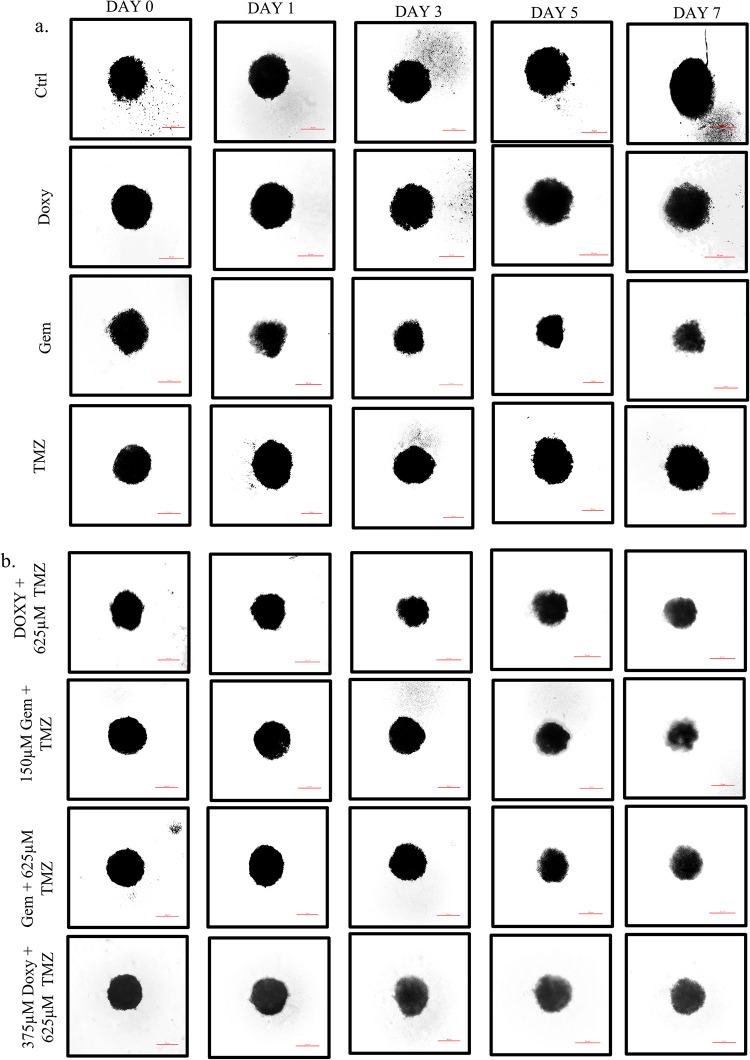

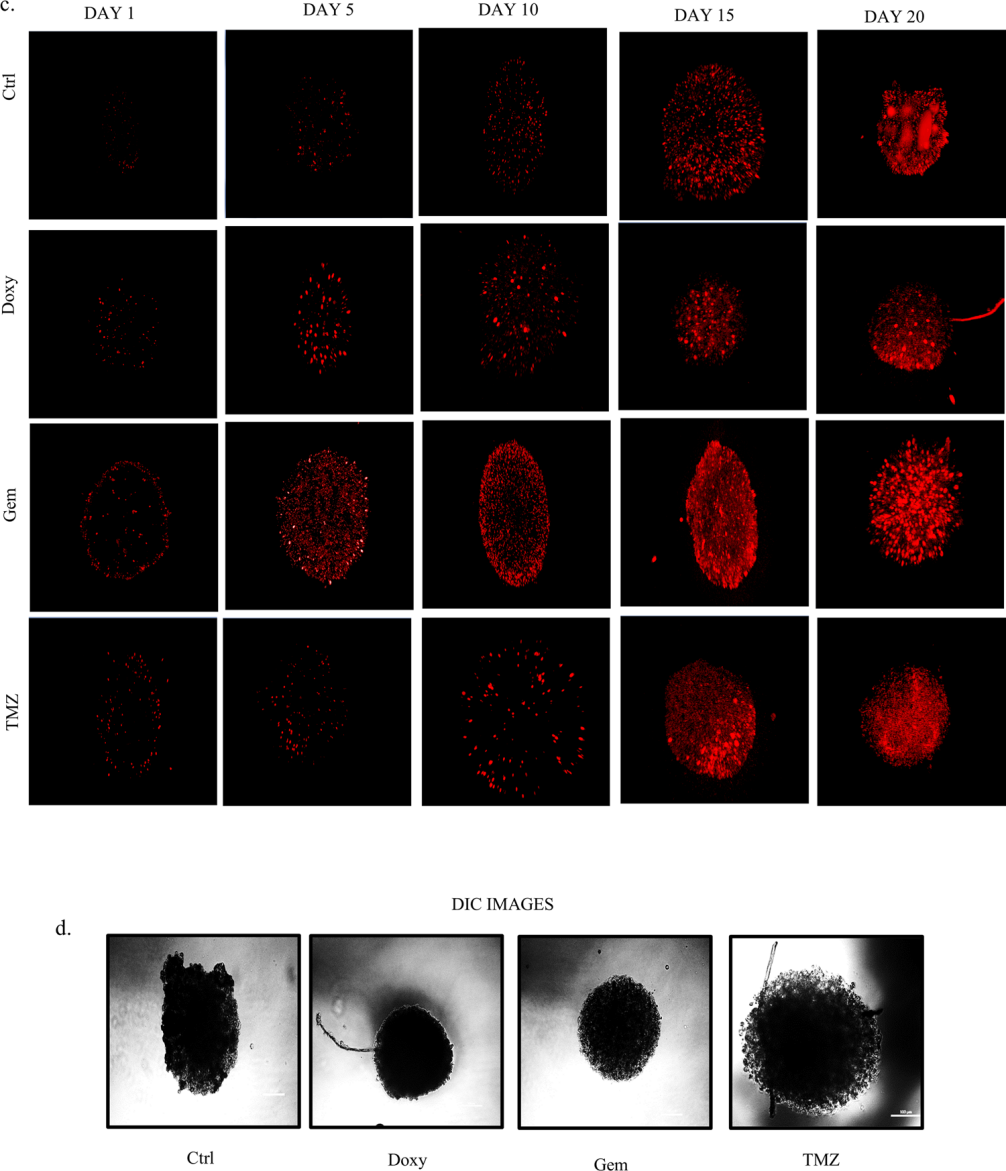
Gemfibrozil and Doxylamine show cytotoxicity in 3-D spheroid cultures of GBM cells: (**a**) Phase contrast images of the 3-D spheroids cultures post-treatment with drugs or drug combinations, (**b**) Microscopic images of 3-D spheroids of Doxy/Gem/ Drugs + TMZ captured after 0, 1, 3, 5, and 7 days respectively. The scale bar represents 20 μm. (**c**) Fluorescent images of 3-D spheroid cultures were captured after 1, 5, 10, 15, and 20 days of Doxylamine and Gemfibrozil treatment and stained with propidium iodide. (**d**) DIC images were captured post-20 days of drug treatment, and the scale bar represents 100 µm.

Since our initial cytotoxicity was analyzed in adherent in-vitro cultures, which have their own limitations, we further validated the GBM inhibitory potential of the drugs through spheroid cultures as well. Recent literature suggests that tumor cells show increased refractoriness to drugs in three-dimensional (3D) states compared to the conventional culture methods, and therefore, validation of drug effects in the 3D systems has become a practice as it better simulates an in vivo milieu than two-dimensional methods practiced. Therefore, it has the potential to serve as a bridge between 2D culture and tests conducted on animals. Interestingly, through phase contrast images captured on days 1, 3, 5, and 7 post-drug therapy, we show a reduction in the size of the spheroids with both the individual treatment with Doxy/Gem or a combination with TMZ, indicating that the drugs are effective not only in two dimensional systems but in 3D as well (Fig. 5A). Importantly, while Gem exposure for 48 h showed a distinct decrease in the spheroid’s diameter, TMZ, on the other hand, failed to show any significant impact. Also, as mentioned earlier, a synergistic effect was observed for Doxy + TMZ and Gem + TMZ after 72 h time period as well (Fig. 5B). The size of each spheroid was measured and a bar graph was ploted representing the change in the spheroid shape with time (Supplementary Fig. 2A,B). Some non-viable cells may remain trapped inside the spheroid culture. To accurately quantify the number of dead cells after treatment, the spheroids were stained with propidium iodide, a dye that only non-viable cells can absorb. With Doxy and Gem cells significant number of cells were found to take up the PI stain even after 5 days of drug treatment. While the control group and TMZ-treated spheroids barely showed any fluorescence activity. During prolonged treatment, the shape of the spheroids began to distort, and even the cells in the control group started to die. This was evident from the red fluorescence stain observed after 15 days of treatment. The shape of the spheroids was also monitored through the DIC images captured after 20 days of treatment. This also confirmed that Doxy and Gem are better alternatives than TMZ, even in 3-D cultures (Fig. 5C–D).

### Doxylamine and Gemfibrozil inhibit autophagic flux in GBM cells

**Fig. 6:**
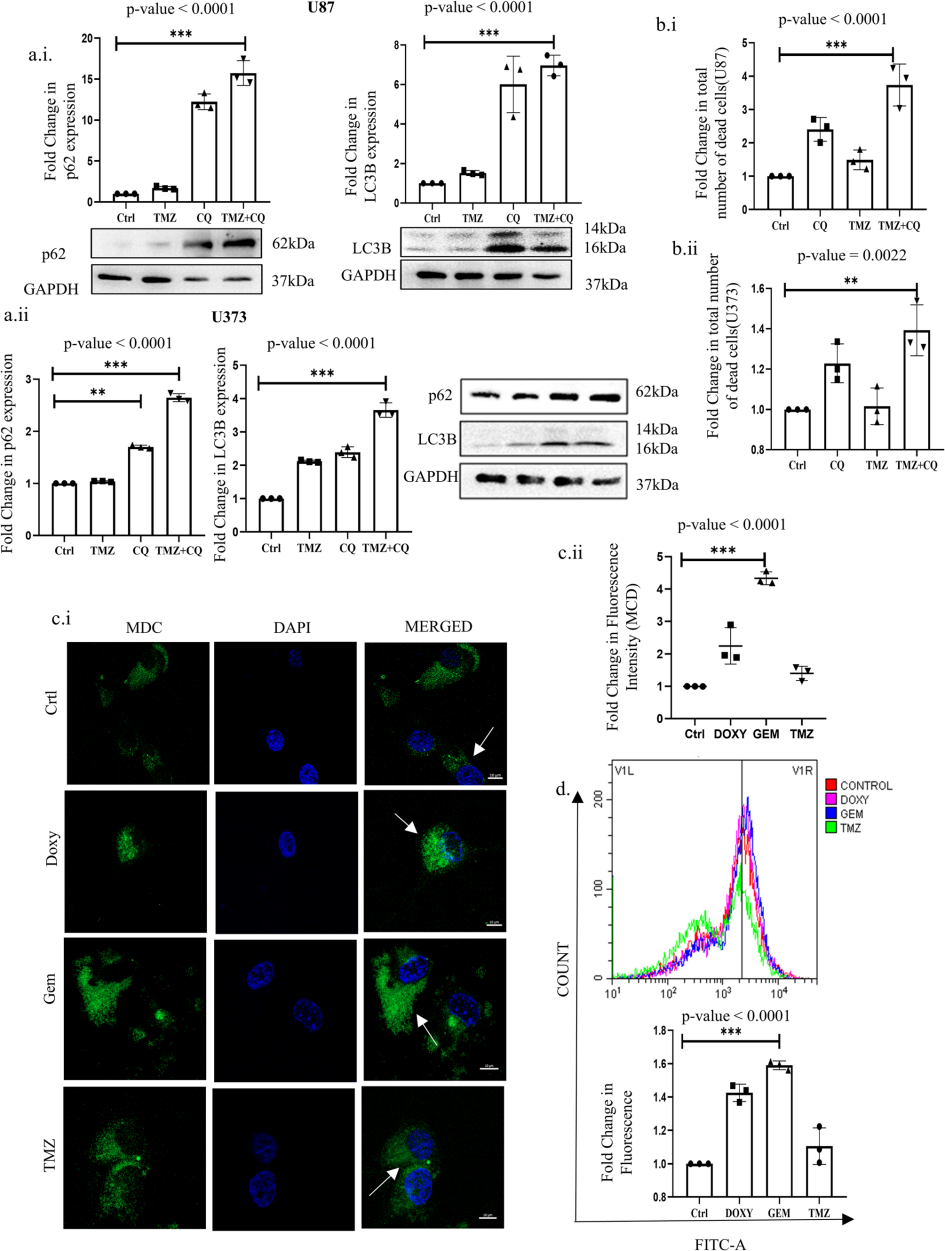

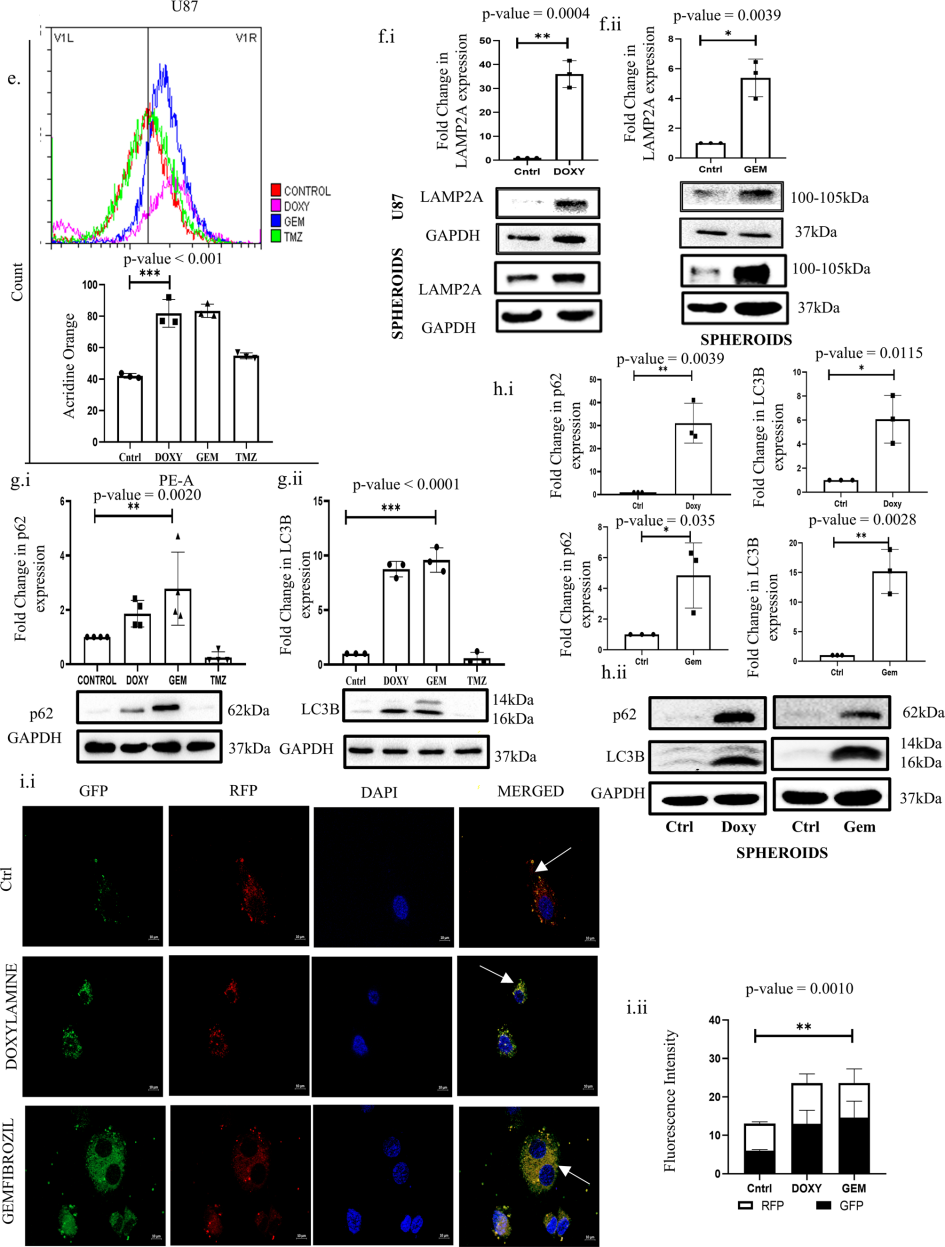
Doxylamine and Gemfibrozil inhibit autophagic flux in GBM cells: (**a**) Immunoblotting showing the expression of p62 and LC3 post-TMZ and TMZ plus CQ co-treatment for 48 h in (**i**) U87 cells and (**ii**) U373 cells, (**b**) Determination of cell death in TMZ and/or CQ (10 μM) treated cells as analyzed through Annexin V-PI staining in GBM cells [(i) U87 and (ii) U373], (**c.i**) Fluorescence intensity of the cells stained with MDC after Doxy, Gem, and TMZ treatment for 48 h. Scale bar represents 10 μm, (**c.ii**) Dot plot representing the fold change of fluorescence intensity plotted, (**d**) Fluorometric analysis of MDC fluorescence in cells treated with each drug for 48 h, (**e**) Flow cytometric analysis representing a shift in AO fluorescence after individual drug treatment, (**f.i**,**f.ii**) Immunoblotting of LAMP2A protein post-Doxy and gem treatment confirming lysosomal accumulation in both 2-D and 3-D U87 cultures, (**g.i,g.ii**) Immunoblots showing the expression of p62 and LC3 in U87 cells after 48 h of drug incubation confirming autophagic flux inhibition, (**h.i**) Immunoblotting of p62 and LC3B of the U87 Spheroid cultures post 5 days of drug treatment showing accumulations of the autophagic proteins, (cropped image of the blot is provided), (**h.ii**) Bar graph representing the expression of the autophagic proteins after Doxylamine and Gemfibrozil treatment to the spheroids confirming inhibition of the autophagic flux, (**i.i**) GFP and RFP fluorescence intensity measurement in the cells transfected with GFP-RFP-LC3 followed by individual drug treatment. The scale bar represents 10 μm. (**i.ii**) Bar graph plotted to represent the fold change in fluorescence. *p*-value ≤ 0.05 is considered significant, and * refers to a *p*-value less than or equal to 0.03, ** refers to a *p*-value less than or equal to 0.002, and *** signifies a *p*-value less than 0.0001 between control and different test groups. All experiments are replicated thrice unless mentioned otherwise.

Our *in-silico* analysis suggests the de-regulation of genes involved in intracellular signaling, like PI3k-Akt and calcium-dependent pathways, intricately linked with cellular homeostatic processes like autophagy. Furthermore, a plethora of existing literature also implicates the importance of autophagy in GBM pathogenesis, and hence, there is a significant relevance in understanding and regulating autophagy in GBM cells. To further authenticate the relevance of autophagy in imparting therapy resistance in GBM cells, we treated U87 cells with TMZ and monitored autophagic flux; thereafter, autophagy was inhibited, and the sensitivity of the cells to TMZ was evaluated. As expected, the U87 and U373 cells showed a drastic accumulation of LC3-BII with TMZ. However, this accumulation could be attributed to either an activation of autophagic flux or inhibition of the fusion of lysosomes and autophagosomes^34^. To confirm the same, the fusion of vesicles was inhibited with Chloroquine (CQ), and we observed an accumulation of both p62 and LC3B-II proteins above only CQ treatment, indicating activation of autophagic flux with TMZ (Fig. 6A). Importantly, analysis of apoptosis in both U87 and U373 cells showed that inhibition of autophagy with CQ also results in increased cell death compared to independent treatments of CQ or TMZ, thus signifying the pro-survival function of autophagy in these cell types (Fig. 6B). Herein, we further planned to determine whether the selected drugs from this study- Doxy and Gem have any role in perturbing the autophagic process in the GBM cells. If so, this can be of immense relevance to GBM therapy. Interestingly, Doxy and Gem boosted autophagosome accumulation, which was evaluated through fluorescence imaging and flow cytometric analysis of MDC green puncta, which is indicative of autophagosome accumulation^35,36^ (Fig. 6C,D). On a similar note, acridine orange dye, which is reported to label acidic vacuoles like lysosomes, also showed a similar trend^37^ (Fig. 6E). Furthermore, immunoblotting of the lysosomal membrane protein marker- LAMP2A also depicted a trend of accumulation, indicating a lysosomal buildup both in U87 cells as well as in the 3-D spheroid cultures (Fig. 6F). The above observations can be resultant of either activation of autophagy or a block of vesicle trafficking leading to disruption of cargo clearance post-exposure to Doxy or Gem. Importantly, the latter proposition was confirmed when we observed an accumulation of both LC3B-II and p62 proteins that are degraded during active autophagic flux (Fig. 6G)^38–40^. Similar results were observed in cells under both 2-D and 3-D culture conditions, strongly confirming the inhibition of autophagic flux following drug treatment (Fig. 6H). In addition, U87 cells transfected with GFP-RFP-tagged LC3B demonstrated an increase in proportional green fluorescence compared to red after Doxy and Gem exposure, indicating autophagy suppression (Fig. 6I)^41^. Our findings, for the first time, indicate that the CNS accumulating drugs (Gem and Doxy) with BBB penetrance have the potential to impede autophagy by preventing autophagosome-lysosome fusion, which has clinical relevance in GBM pathogenesis and therapy resistance.

### Doxylamine and Gemfibrozil synergize with TMZ or CQ, inducing enhanced cell death

**Fig. 7:**
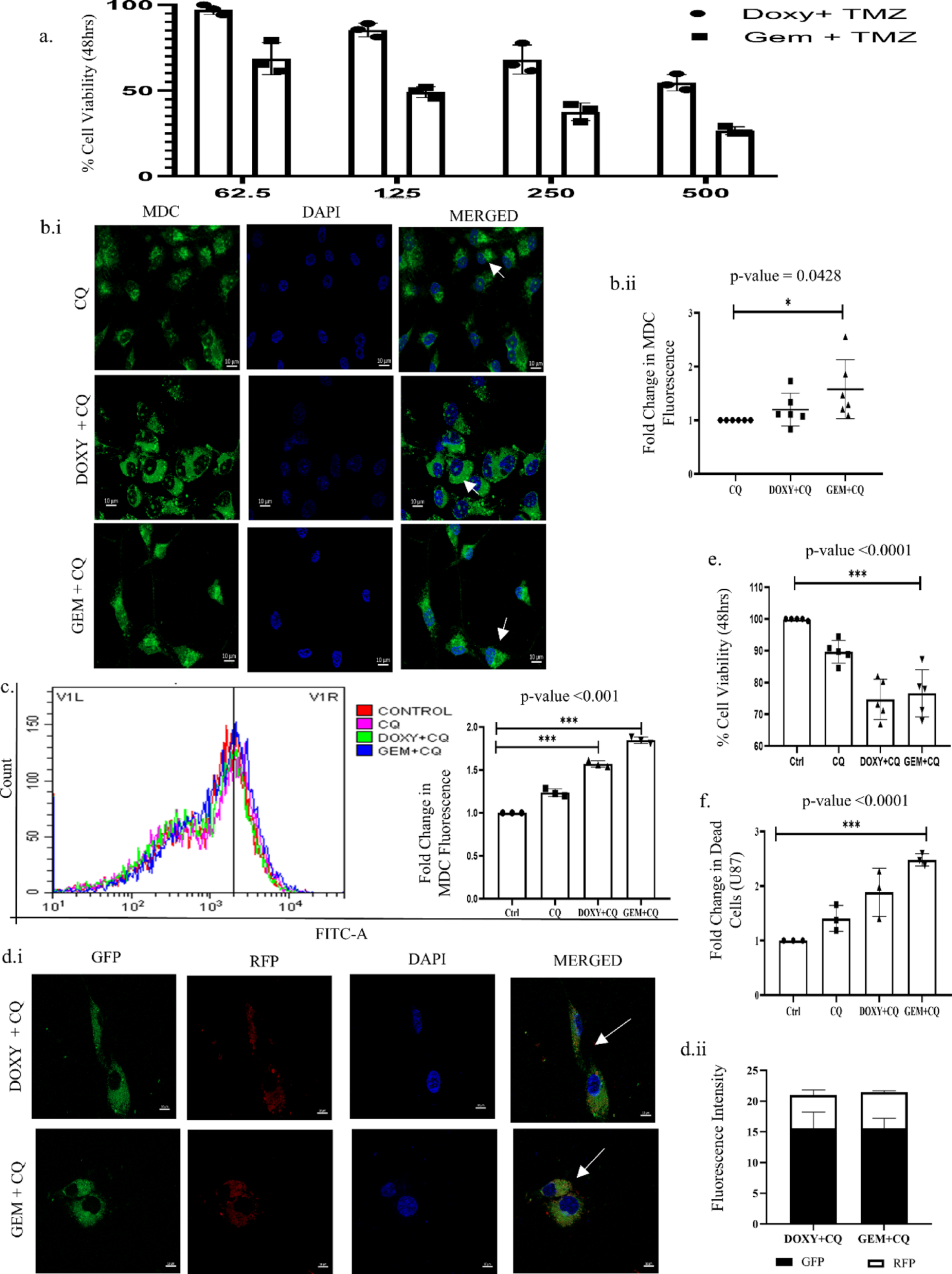
Doxylamine and Gemfibrozil synergize with TMZ or CQ, inducing enhanced cell death: (**a**) MTT assay performed to determine the percent survival of U87 cells when co-treated with Doxy or Gem and/or TMZ for 48 h, (**b.i**) Fluorescence intensity of MDC staining visualized under the microscope to confirm the increased accumulation of autophagosomes post-treatment in U87 cells. The scale bar represents 10 μm. (**b.ii**) Dot plot representing the change in fluorescence intensity, (**c**) Fluorimetric analysis of the MDC stain post-treatment. A bar graph was plotted, (**d.i**) GFP and RFP intensity of the cells transfected with GFP-RFP-LC3 vector post-co-treatment with CQ and drugs. Scale bar represents 10 μm, (**d.ii**) Bar graph plotted to represent the change in GFP to RFP fluorescence, (**e**) MTT assay performed to confirm the cytotoxicity of the drugs when co-treated with an autophagic inhibitor, CQ, (**f**) Analysis of the total number of dead cells post CQ and CQ plus drugs treatment for 48 h by AnnexinV-PI assay. *p*-value less than or equal to 0.03, ** refers to a *p*-value less than or equal to 0.002, and *** signifies a *p*-value less than 0.0001 between control and different test groups. All experiments are replicated thrice unless mentioned otherwise.

To understand whether these drugs can synergize with the existing drug used for GBM therapy, we exposed GBM cells to various concentrations of Gem or Doxy along with TMZ. This resulted in enhanced cytotoxicity as analyzed through MTT assay (Fig. 7A). Furthermore, these drugs were found to enhance the autophagy inhibitory effects of CQ as well. An increased accumulation of MDC fluorescence was observed in U87 cells after treatment with CQ and Doxy/Gem (Fig. 7B,C). Similar results were also obtained with the transfected GFP-RFP-LC3 vector (Fig. 7D). Importantly, both MTT assay and AnnexinV/PI staining showed an increase in cell death upon co-treatment of Gem/Doxy with CQ (Fig. 7E,F). Our findings indicate that autophagy acts as a pro-survival strategy in the GBM cells studied and contributes to TMZ resistance, and Doxy and Gem can be potent alternatives to TMZ to sensitize these cells.

### Gemfibrozil and Doxylamine generate reactive oxygen species (ROS) to induce cytotoxicity

**Fig. 8:**
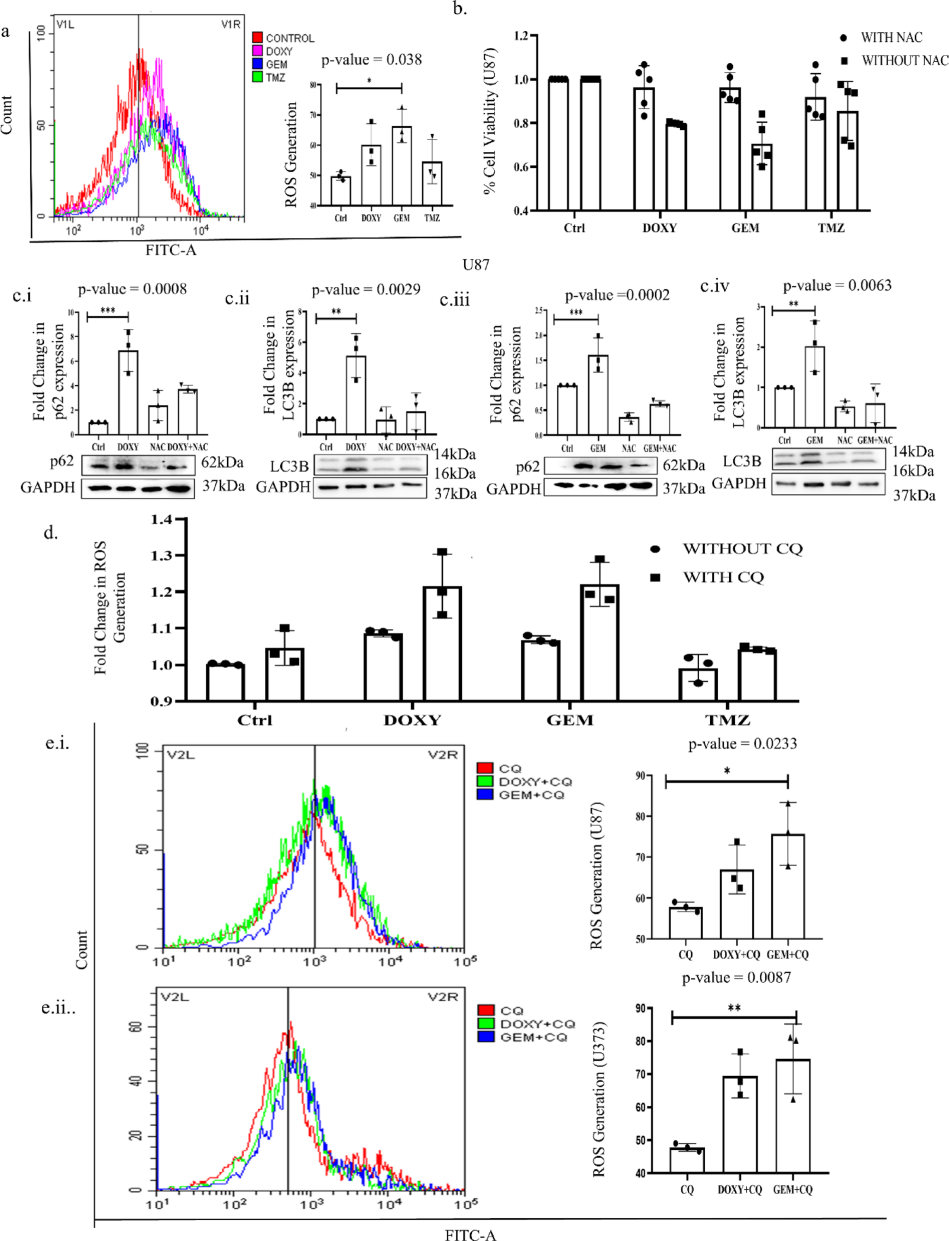
Gemfibrozil and Doxylamine generate reactive oxygen species (ROS) to induce cytotoxicity: (**a**) Fluorimetric analysis of ROS generation post-drug treatment for 48 h, (**b**) Analysis of cell death with and without NAC treatment prior to Doxy and Gem by MTT assay, (**c.i.-iv.**) Western blotting analysis of p62 and LC3 co-treated with NAC and Doxy or Gem treatment for 48 h in U87 cells, (cropped image of the blot is provided), (**d**) Fluorimetric analysis of ROS generation of cells co-treated with CQ and Doxy or Gem treatment for 48 h, (**e**) Fluorimetric analysis of ROS generation of the cells treated with CQ and Doxy or Gem treatment for 48 h in (i) U87 cells and (ii) U373 cells. * indicates the significant difference between control & different groups, calculated using the student’s t-test and one-way or two-way ANOVA with Bonferroni post-test. *p*-value ≤ 0.03 is considered significant. Unless otherwise stated, experiments are replicated three times.

Autophagy has been implicated in regulating cellular redox balance. Since the drugs screened in our study showed an autophagy inhibitory potential, we planned to explore their effects on ROS as well. Interestingly, we observed a substantial increase in ROS production in the U87 cells after the drug treatment; in fact, the amount of ROS generated was even more than in TMZ treatment alone (Fig. 8A). To further confirm if the generation of ROS has any correlation with cell viability, we quenched ROS with N-acetyl cysteine (NAC), and an increase in cell viability was observed when ROS was quenched (Fig. 8B). To understand if ROS generation is modulating autophagy in the cells after the drug treatment, the expression of p62 and LC3 was checked. NAC resulted in the reversal of Gem/Doxy-induced inhibition of autophagic flux (Fig. 8C). On the contrary, an increased ROS generation was observed after co-treatment of CQ with the drugs both in U87 and U373 cells, confirming that autophagy inhibition results in the generation of ROS, which leads to the observed cytotoxic effects (Fig. 8D,E). Overall, our study shows that Gem/Doxy, through their autophagy inhibitory potential, disturbs the cellular redox balance and imparts a cytotoxic effect on the GBM cells studied.

## Discussion

The development of novel drug formulations faces significant challenges, which includes high research and development costs, elevated failure rates, and study on limited patient populations, which collectively reduce the incentive for pharmaceutical companies to invest in such endeavours. Consequently, the repurposing of already-approved drugs, particularly those with established safety profiles, offers a promising and cost-effective alternative for therapeutic development^42–45^. This strategy is especially valuable for treating complex conditions like glioblastoma (GBM), where BBB presents a major obstacle to effective drug delivery. Repurposed drugs with demonstrated BBB permeability can have the potential to expedite clinical translation, provided their efficacy is validated through rigorous preclinical and clinical evaluation. In this regard, cancer treatment has increasingly benefited from drug repurposing, particularly due to the challenges associated with developing novel therapeutics, as described above. There are several existing examples of drug repurposing in cancer- for instance, Li et al*.* developed a pipeline to repurpose kinase inhibitors against various cancers^46^; similarly, Touhey et al*.* demonstrated that inhibition of MRP-1 by indomethacin and its analogues can enhance the cytotoxicity of several chemotherapeutic agents at non-toxic concentrations^47^. Repurposing has also been explored for GBM, for example, Andrino et al*.* optimised the TISMAN (Transcriptomics-Informed Stoichiometric Modelling and Network) framework to identify pre-existing drugs like Afuresertib and Taxifolin^48^. However, Taxifolin’s limited stability and poor bioavailability restricted its clinical utility, necessitating advanced delivery systems, detailed pharmacokinetic profiling and search for alternate therapies^49^. Further, Lun et al*.* identified Disulfiram as a potential alternative to TMZ in the presence of copper gluconate through high-throughput in vitro screening^50^. However, a more recent study revealed that combining Disulfiram with copper during chemotherapy can significantly increase the incidence of serious adverse events in GBM patients. Similarly, Vorinostat, a histone deacetylase (HDAC) inhibitor, has shown antitumor effects in GBM models by upregulating pro-apoptotic genes and downregulating cell cycle-related proteins^51,52^. It has been found to effectively cross the blood–brain barrier and exhibit some clinical benefit in recurrent GBM, though common side effects include thrombocytopenia and fatiguev^53^. These safety concerns, poor bioavailability, along with limited clinical evidence, currently constrain therapeutic application of repurposed drugs against GBM warranting further exploration^54^. Therefore, as a gateway to identification of effective brain peneterative therapeutics against GBM several studies have now explored the use of anti-depressants or neuro-regulatory drugs. If found effective, these drugs can act as a safer alternative with proven brain availability. In this regard, tricyclic antidepressants like amitriptyline, clomipramine, and doxepin were found to inhibit the PI3K/Akt/mTOR pathway, reducing GBM stemness, and modulate cell plasticity^55^. Similarly, selective serotonin reuptake inhibitors (SSRIs) such as sertraline, citalopram, escitalopram, and paroxetine were found to promote apoptosis in GBM cells by disrupting actin formation and modulating signalling pathways like FAK, Akt, and mTOR, while also increasing calcium influx into mitochondria^56,57^. However, the translation of these drugs to the clinics for GBM treatment has not been smooth and needs more deliberation in terms of mechanistic analysis of their efficacy or clinical trials in large patient cohort. In this context, drugs with a well-established clinical safety record, known ability to cross the BBB and with potential to address the disease-associated genetic aberrations can be of particular interest for repurposing against GBM. Inculcating a genetic approach to the CNS-penetrant drugs can be an ideal option for disease reversal strategies^58,59^. Herein, using molecular signatures derived from the cancer genome atlas (TCGA), we identified key hub genes associated with GBM pathogenesis, including CAMK2A, FOXM1, SYN1, CALM1, GRIN2A, NEFL, GABRA1, IL6, ATP2B2, and SNCB^60^. These genes were used to query the connectivity map database, leading to the identification of potential modulators with therapeutic relevance. Our in silico analyses screened existing FDA-approved drugs and identified compounds like, Valproic Acid (a histone deacetylase- HDAC inhibitor), Gemfibrozil (a lipoprotein lipase inhibitor and PPAR agonist), and atypical psychotropic drugs like Doxylamine (an antihistamine) and Diprophylline (an adenosine receptor antagonist) as probable potential therapeutic alternatives for GBM. These drugs were further evaluated for their cytotoxic effects against GBM and their ability to disrupt molecular pathways central to GBM progression. Given that GBM remains one of the most lethal and treatment-resistant cancers, we observed a high IC_50_^61–63^ value for the standard drug- TMZ when compared to Gemfibrozil, a known PPAR agonist^64–66^ or Doxylamine^67,68^, an antihistamine. These findings suggest that Gemfibrozil and Doxylamine may possess superior anti-tumour efficacy against GBM cells in vitro and could serve as potential alternative candidates for further preclinical development. Importantly, the other shortlisted drugs- Valproic Acid^67,69,70^ or Diprophylline^30^, failed to induce any measurable cytotoxicity in the GBM cells studied even after 72 h of drug exposure; the IC_50_ values could not be determined at the maximum time point studied.

Furthermore, to gain mechansitic insights into functioning of Gem and Doxy, we investigated alteration in autophagy, a key cellular homeostatic process, which has been implicated in the development of TMZ resistance in GBM. For example, Yun et al*.* demonstrated that TMZ resistance can be driven by activation of the Wnt/β-catenin signalling pathway through autophagy induction. Similarly, cytoprotective autophagy mediated by YAP has been linked to enhanced malignancy in GBM^71,72^. Consequently, targeting autophagic flux has emerged as a promising approach, with agents like chloroquine- an autophagy inhibitor, currently in phase III clinical trials for GBM treatment, underlining the therapeutic relevance of this pathway^73,74^. As part of our study, we therefore assessed the effects of the in silico identified drug candidates on autophagy regulation and associated cytotoxicity. Interestingly, we discovered that both Doxy and Gem significantly impaired GBM cell survival by inhibiting autophagic flux. This disruption led to an increased intracellular ROS accumulation and subsequent cell death. Similar anti-cancer activity via autophagy inhibition has previously been reported for antihistamines such as Deptropine^75^. However, our study is the first of its kind to demonstrate the anti-autophagic action of BBB-penetrant drugs like Doxy and Gem and their associated cytotoxic effects on GBM cells.

Despite promising results, there are several challenges that hinder the clinical use of repurposed drugs for GBM. In this study, combining in silico predictions, in vitro assays, and 3D culture models, our findings support the repurposing of two CNS-accumulating drugs as potential GBM therapeutics. Both agents exhibited strong BBB permeability, inhibited autophagy, and sensitized GBM cells to cytotoxic stress. While the results are promising, effect on GBM stem cells, further preclinical studies and validation are warranted to advance these compounds toward clinical trials for GBM treatment.

## Materials and methods

### GBM patient data and gene expression analysis

The transcriptomic data of the GBM patients and normal control were obtained and extracted from NCBI’s Gene Expression Omnibus (GEO)^76^, with accession ID GSE2223^77^, and GSE4290^78^. These datasets contain a total of 127 glioblastoma patient samples and 27 non-tumour brain samples. The transcriptomic data were analysed using the GEO2R tool under NCBI. Genes with adjusted *p*-values at or below 0.05 and with log2(FC) value (fold change) ≥  + 2 or ≤ -2 (with respect to control) were considered to be differentially expressed.

### Functional annotation and pathway analysis of differentially expressed genes in GBM

Significantly dysregulated genes were subjected to functional annotation of GO and KEGG pathway enrichment. Web-based DAVID tool^79,80^, KEGG mapper^81–83^ and ClueGO software^84^ were used for integrative analysis to comprehensively observe the DEGs involved in the GO terms and pathways. The results from DAVID were imported to GOplot in R Studio to visualise the functional enrichment of the top DEGs. The study of molecular function, biological process, cellular component and enrichment of pathways analysis was conducted for DEGs, and *p*-values ≤ 0.05 were considered to be significant. The significantly DEGs associated with all three GO functional categories were termed as ‘key genes’. The Supplementary Fig. 3 depicts the detailed steps of functional annotation and pathway analysis of DEGs.

### Protein–Protein interaction network

The physical and functional associations among the proteins of DEGs were assessed using the STRING tool^85^. The minimum required interaction score was set to a confidence of 0.4. PPI integrated networks were then imported and visualised with Cytoscape software. The highly connected hub genes were identified using cytoHubba, which is one of the applications of Cytoscape, and it ranks nodes in a network by their network features^86^.

### Mining of disease signature reversal drugs

The connectivity map (CMap) database (https://portals.broadinstitute.org/cmap/) was utilised for the retrieval of FDA-approved drugs that can have the potential to reverse the disease signature of GBM^87^ and ability to modulate autophagy. Broad Institute’s Connectivity Map, or CMap, is a tool for analyzing large perturbation datasets with the goal of speeding up the creation of new medicines or the repurposing of existing therapeutics. Drugs exhibiting a negative connectivity score, reflecting their capacity to revert back disease-associated gene activity, were selected as potential therapeutic candidates. The negative connectivity score was selected as a primary criterion for drug screening because it provides a strong indication of a drug’s ability to reverse the gene expression signature associated with GBM. The shortlisted drugs are then screened for their BBB crossing potential based on their total polar surface area (TPSA), molecular weight and partition coefficient. The greater the lipophilicity and the smaller the molecular weight, more are the chances of a drug crossing the BBB effectively. Using STITCH, a network of the drugs (obtained from Connectivity Map) and the hub genes (identified through STRING) was formed. STITCH (http://stitch.embl.de/, STITCH)^27^ is a tool that combines the information of interactions obtained from metabolic pathways, crystal structures, binding experiments, and drug–target relationships to provide an overview of the potential effects of the chemical on its interaction partners.

### Drug perturbation signatures in GBM cell lines

The genomics of drug sensitivity (https://www.cancerrxgene.org/) in the cancer portal was used to obtain drug perturbation data in GBM cell lines. A list of drugs that were tested on the GBM cell line, along with their z-scores, was extracted. All the protein targets and genes perturbed by those drugs were obtained, and a PPI network employing the STRING database was generated. The list of protein targets of all those drugs was downloaded, and a PPI network was created with the STRING database.

### Chemicals and reagents

The drugs- Doxylamine (#D3775), Gemfibrozil (#69518), Chloroquine (CQ, #C6528) and Valproic Acid (#P4543) were procured from Sigma Aldrich. In addition, Protease Inhibitor Cocktail (PIC, #P8340), RIPA Buffer (#R0278), Propidium Iodide (PI, #P4864) and Monodansylcadaverine (MDC, #D4008) were also obtained from Sigma. Temozolomide (#85622-93-1) and Diprophylline (#479-18-5) were purchased from TCI Chemicals. From Thermo Fisher Scientific we procured N-acetyl- L-cysteine (NAC, #47,866), 3-[4, 5-dimethylthiazol-2-yl]-2,5-di-phenyltetrazolium bromide (#33,611; MTT); Crystal Violet (CV, #28376) were obtained from SRL^88–90^ AnnexinV binding buffer (#V13246), FITC conjugated AnnexinV (#A13199), Enhanced Chemiluminescence (ECL, #32106), Lipofectamine-3000 (#L3000-001), Antifademountant (4ʹ-6-diamidino-2-phenylindole, #P36962) were purchased from Invitrogen. Anti-GAPDH (#sc-365062) was procured from Santa Cruz Biotechnology, and primary antibodies such as anti-p62 (#D1Q5S), anti-LC3B-II (#D-11 3868S), anti-PCNA (#D3H8P), and anti-Caspase-3 (#D3R6Y 14220), and secondary antibodies- anti-rabbit (#7074P2) and anti-mouse (#7076S) were procured from Cell Signaling Technology (CST), and anti-LAMP2 (#ab18528) from Abcam. Plasmid GFP-RFP-LC3 was generous gift from Dr. Sovan Sarkar (associated with the University of Birmingham as a Birmingham fellow)^91^

### MTT assay

Cytotoxicity analysis of FDA-approved selected drugs on U87 (purchased from NCCS; Pune, India) and U373 (purchased from Sigma Aldrich) glioblastoma cell line was performed for 24, 48 and 72-h time points using the procedure described in detail elsewhere^88^ which was then compared with Temozolomide (TMZ), a standard drug against Glioblastoma. Briefly, the formation of formazan crystals by live cells after the addition of MTT was monitored and measured by analyzing optical density.

### Crystal violet staining

Cells seeded in 6 well plates were exposed to drugs and then checked for crystal violet fluorescence to confirm drug activity on U373 and U87 cells. Triarylmethane dyes like Crystal violet stains bind specifically to ribose-type molecules like DNA in the nuclei of living cells. We treated the cell line with the IC_50_ values for every drug and observed the long-term effects of the medicines during 48 and 72 h of incubation. After 72 h, crystal violet stain was added to the cells. The stains taken up by the viable cells were then visualized and captured using a Zeiss Primovert microscope to determine how many cells were still alive^92^.

### 3-D spheroid culture

The cells were trypsinized, the single-cell solution was mixed with methylcellulose, and the final 10,000 cells per 10µl were maintained to form a sphere. 10µl of the cell mixture was then added dropwise on the lid of the 10 cm petri plate and was kept in an inverted position, and 10 ml of PBS was added to the plate to provide moisture to the cells. The Spheroids were allowed to take their shape, and thereafter, the spheroids were transferred to a round-bottomed 96-well plate, and the drugs were added. At regular intervals phase contrast images were captured to determine the change in shape of the spheroids as well as the spheroids were stained with Propidium Iodide to properly determine the number of dead cells trapped inside the spheroids and the fluorescence intensity was captured under Microscopy (Zen 3.2 Blue edition). Post 5 days of drug treatment, the protein was extracted from the spheroids and Immunoblotting was performed to determine the expression of different proteins.

### Cell viability study by Annexin V

For this study, cells were seeded in 6 cm dishes at 6 × 10^5^ cells per dish cell density. To determine the total number of dead cells, U87 and U373 cells are stained with Annexin V and PI following the procedure described elsewhere^88^. Briefly, selected drug treatment was given to the cultured cells and incubated for 48 h at 37 °C followed by flow cytometric (Beckman Coulter) analysis of apoptosis with AnnexinV-PI. The CytExpert software was used for acquired data analysis.

### MDC staining

The U87 cells seeded on coverslips were treated with specific concentrations of drugs and thereafter incubated for 48 h. The media was then aspirated, washed with PBS, and then live cells were treated with MDC, which stains the autophagic vacuoles^90^. U87 cells were incubated for 30 min with MDC, and then the coverslips were washed and fixed with 4% Paraformaldehyde (PFA). Cells were thereafter mounted with counterstain DAPI and visualized under a microscope (quantitation was done by ZEN 3.2 Lite software).

### Immunoblotting

Cells treated with drugs were harvested in RIPA buffer followed by protein estimation by Bradford assay. An equal amount of protein loaded in polyacrylamide gels was transferred to the polyvinylidene fluoride (PVDF) membrane, followed by blocking with skimmed milk. The probing and re-probing of blots were performed with primary antibodies (dilution 1:1000), and detection was carried out on ChemiDoc (Bio-Rad) using Enhanced Chemiluminescence (ECL). As loading control, GAPDH and total Histone (dilution 1:2000) were used. When necessary, blots were cut to probe with various antibodies to determine protein expression of distinct molecular weights^93^.

### Acridine orange staining

Lysosomal permeabilisation was measured by acridine orange. Cells after specific treatment were extracted, centrifuged (10 min), rinsed in PBS, and resuspended in a fresh medium. Acridine Orange (0.5 μg/mL) was applied and incubated in dark for 20 min. After PBS washing, cells were resuspended in a fresh medium, and data was acquired in a flow cytometer (Beckman Coulter). CytExpert software was used to quantify the shift in fluorescence activity, and bar diagrams were plotted.

### Flow cytometric analysis of autophagic vacuoles

Cells at a density of 6 × 10^5^ cells/well plated in a 6-well plate were exposed to a specific treatment, incubated with MDC stain in the dark for 20–30 min, and thereafter trypsinized. The final pellet of the cells was resuspended in PBS, and samples were finally acquired with the help of the Cytoflex flow cytometer. The fold change in fluorescence is represented.

### Autophagy analysis with GFP-RFP LC3B

The GBM cells were seeded on coverslips and were allowed to gain their desired morphology. The cells were then trasnfected with GFP-RFP tagged LC3B plasmids to understand the better expression of the protein after the drug treatment. After 6 h of transfection, the cells were exposed to IC_50_ values of the drugs and were incubated for 48 h, washed, fixed with 4% PFA, permeabilized with 0.1% Triton X, mounted with DAPI, and visualized under the fluorescent microscope.

### Intracellular reactive oxygen species (ROS) estimation

Cells were seeded in 96-well plates at a density of 8 × 10^3^ cells/well. The cells were pre-treated (1 h) with the ROS scavenger- NAC (10 mM)^94^. After a brief PBS wash, the cells were incubated in DCFH-DA (10 µM; 45 min). Fluorescence was measured at 485 nm excitation and 530 nm emission in a microplate reader (Fluoroskan Ascent).

### Statistical analysis

Mean ± SD was used to represent all the experimental values. Unpaired t-tests were used to compare two groups; one-way ANOVA and two-way ANOVA were used to compare multiple groups without or with having different experimental conditions. Graph Pad Prism 8 was used for analysis. To perform the multiple comparisons amongst the data sets, we used the Bonferroni method. If the *p*-value is < 0.05, then the data is considered statistically significant. * represents the *p*-value less than or equal to 0.03, ** represents the *p*-value less than or equal to 0.002, and *** represents the *p*-value less than 0.0001^95^.

## Electronic supplementary material

Below is the link to the electronic supplementary material.

Supplementary Material 1

Supplementary Material 2

Supplementary Material 3

Supplementary Material 4

Supplementary Material 5

## Data Availability

The transcriptomic data from GBM patients analysed during the current study are available in the GEO repository (https://www.ncbi.nlm.nih.gov/geo/) with accession numbers GSE2223 and GSE4290.
