# Supplementary figures and images for: Repurposing of CNS accumulating drugs Gemfibrozil and Doxylamine for enhanced sensitization of glioblastoma cells through modulation of autophagy
Source: Sci Rep. 2025 Jul 1;15:20560. doi: 10.1038/s41598-025-05054-5 (PMC12219411; doi:10.1038/s41598-025-05054-5)

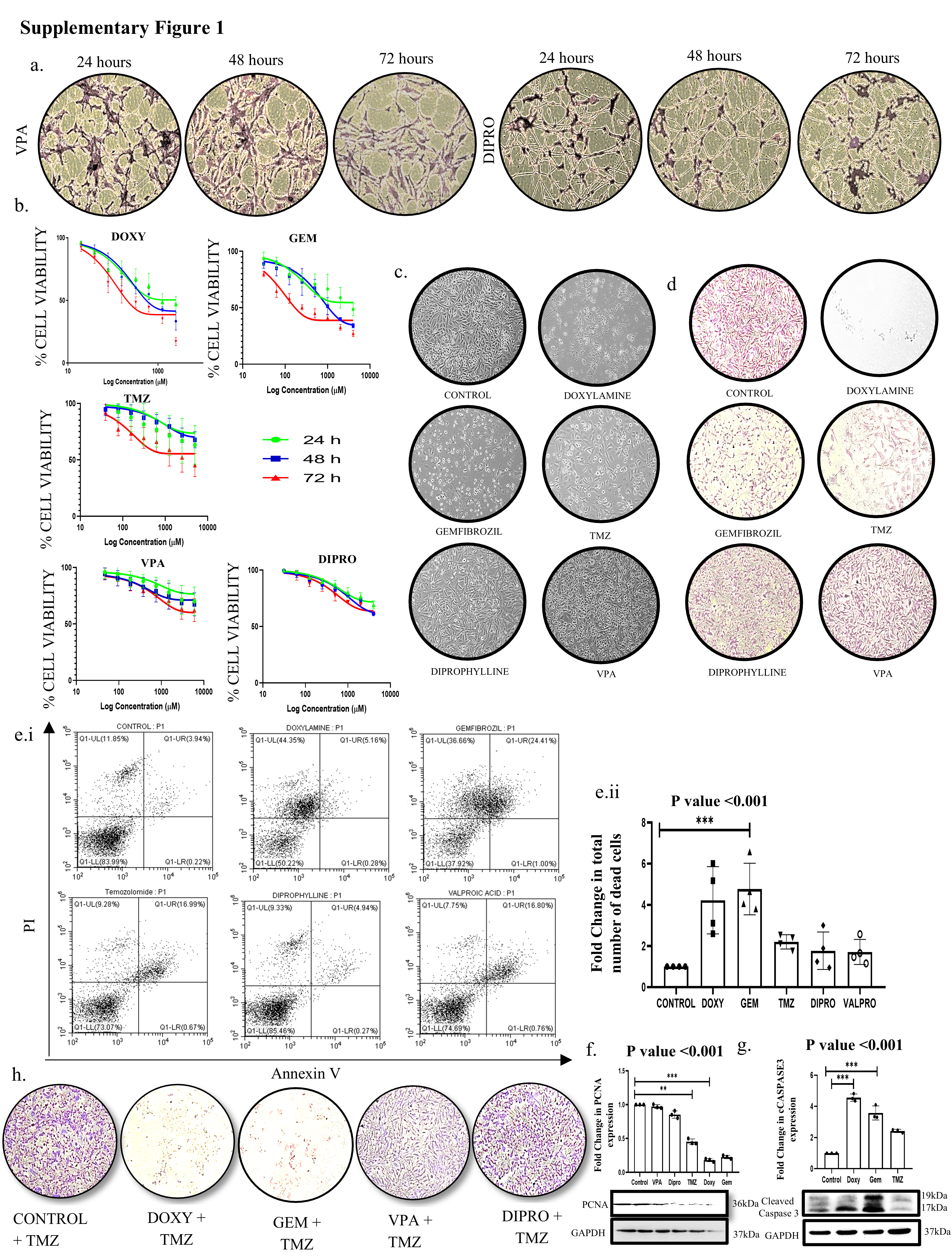

Supplement: Supplementary file 1 — Supplementary Material 1 [file 41598_2025_5054_MOESM1_ESM.tif]

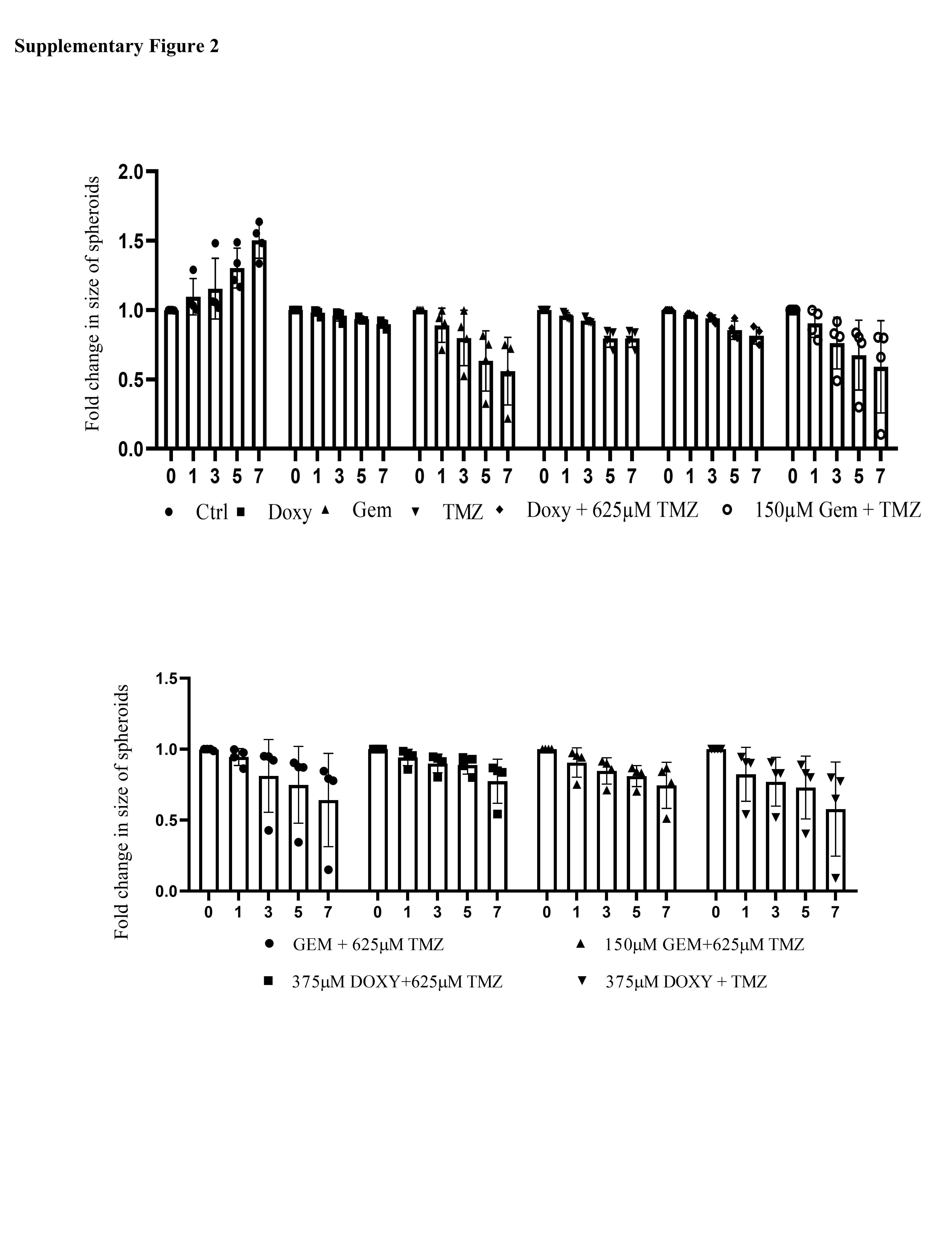

Supplement: Supplementary file 2 — Supplementary Material 2 [file 41598_2025_5054_MOESM2_ESM.tif]

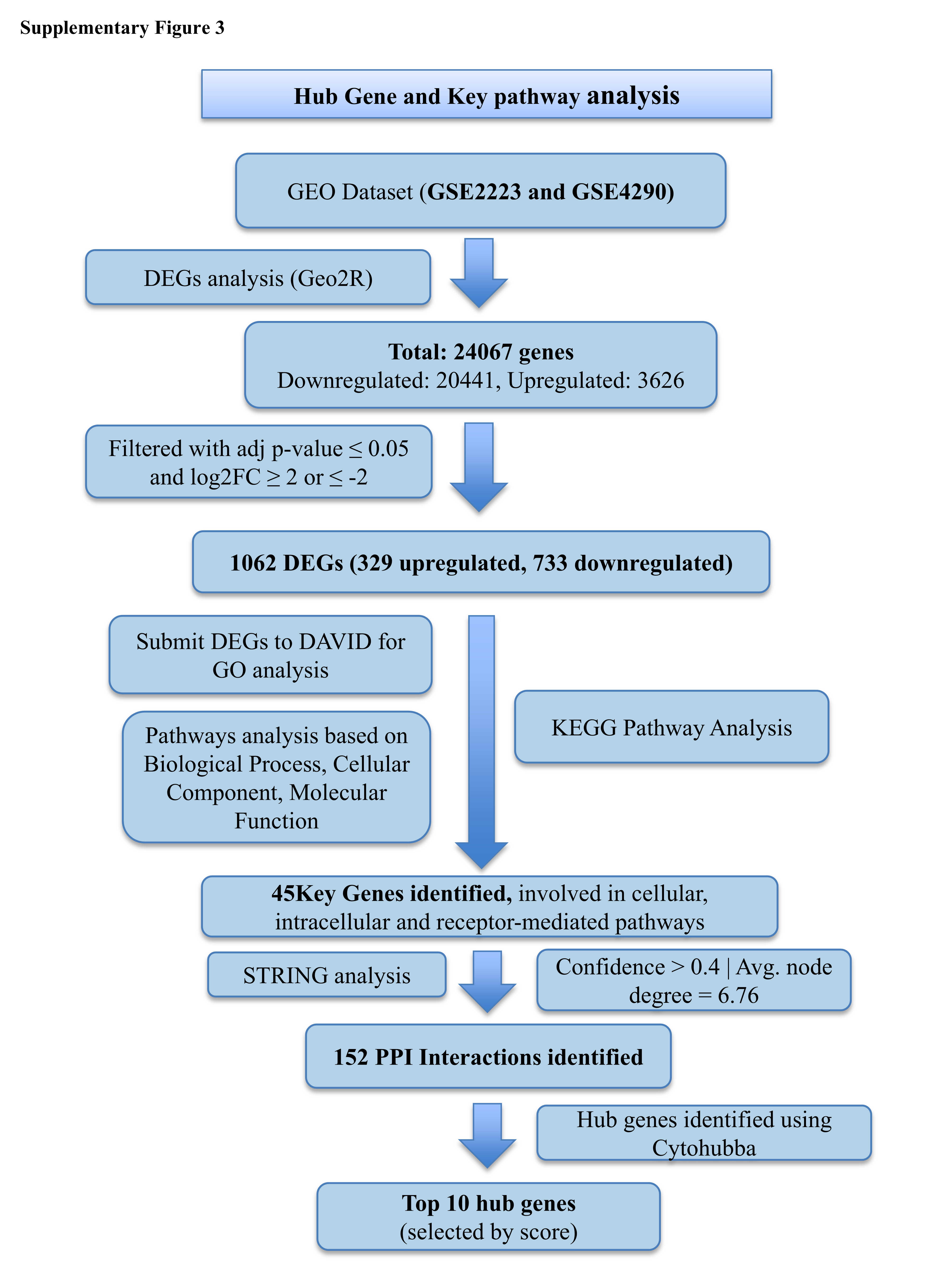

Supplement: Supplementary file 5 — Supplementary Material 5 [file 41598_2025_5054_MOESM5_ESM.tif]
