# Supplementary material for: Repurposing of CNS accumulating drugs Gemfibrozil and Doxylamine for enhanced sensitization of glioblastoma cells through modulation of autophagy
Source: Sci Rep. 2025 Jul 1;15:20560. doi: 10.1038/s41598-025-05054-5 (PMC12219411; doi:10.1038/s41598-025-05054-5)

Cell Line: U87
Treatment: Doxylamine, Gemfibrozil, TMZ, VPA and Diprophylline
Timepoints:24 hours and 48 hours
Experiment: Western Blotting of PCNA
Software Used: Image Lab and Image J

Figure 3.c.i

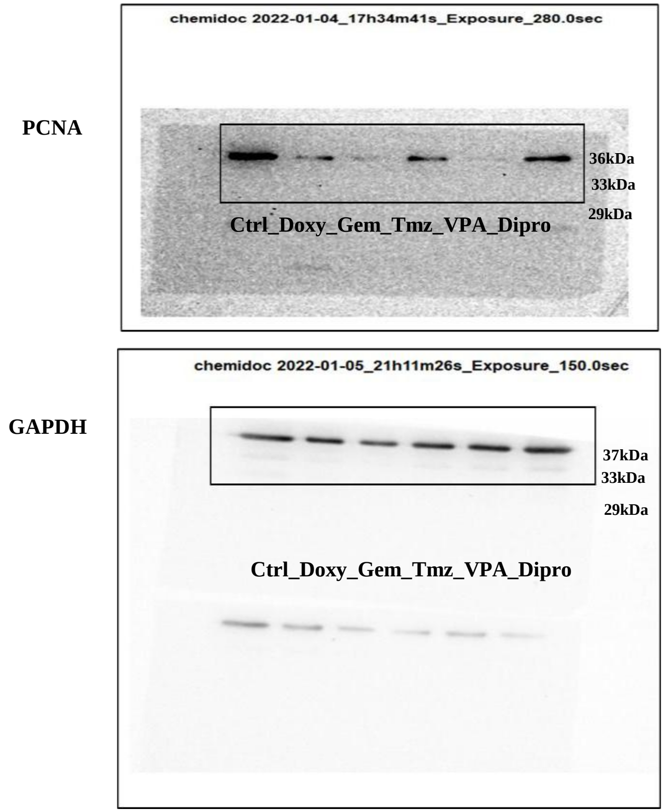

Figure 3.c.ii.

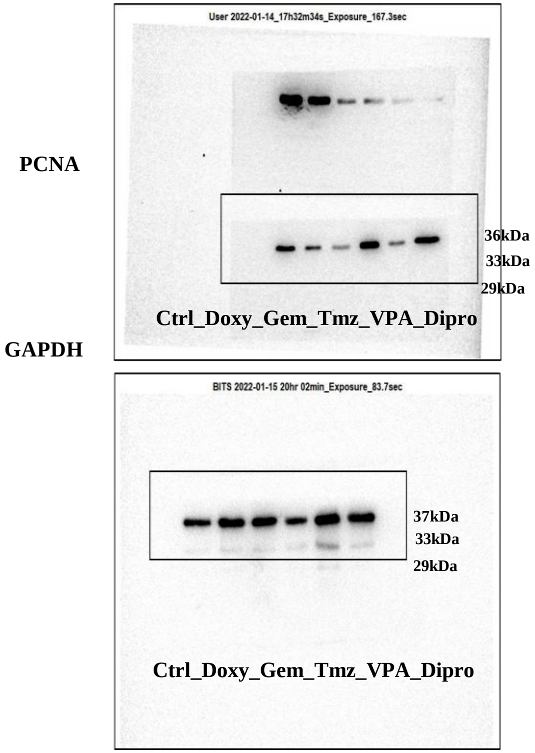

Cell Line: U87
Treatment: Doxylamine, Gemfibrozil, TMZ
Timepoints:48 hours
Experiment: Western Blotting of cCASPASE3 Software Used: Image Lab and Image J

Figure 3. d.

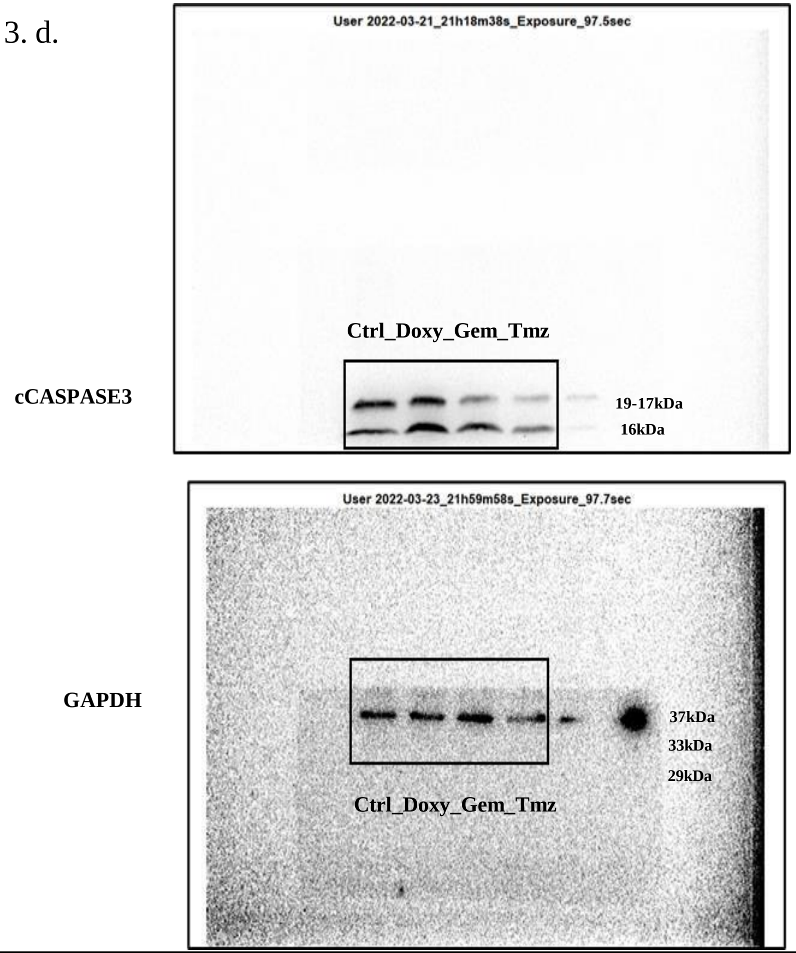

Figure 5.a.i

Cell Line: U87
Treatment: TMZ and CQ
Timepoints:48 hours
Experiment: Western Blotting of p62 and LC3B
Software Used: Image Lab and Image J

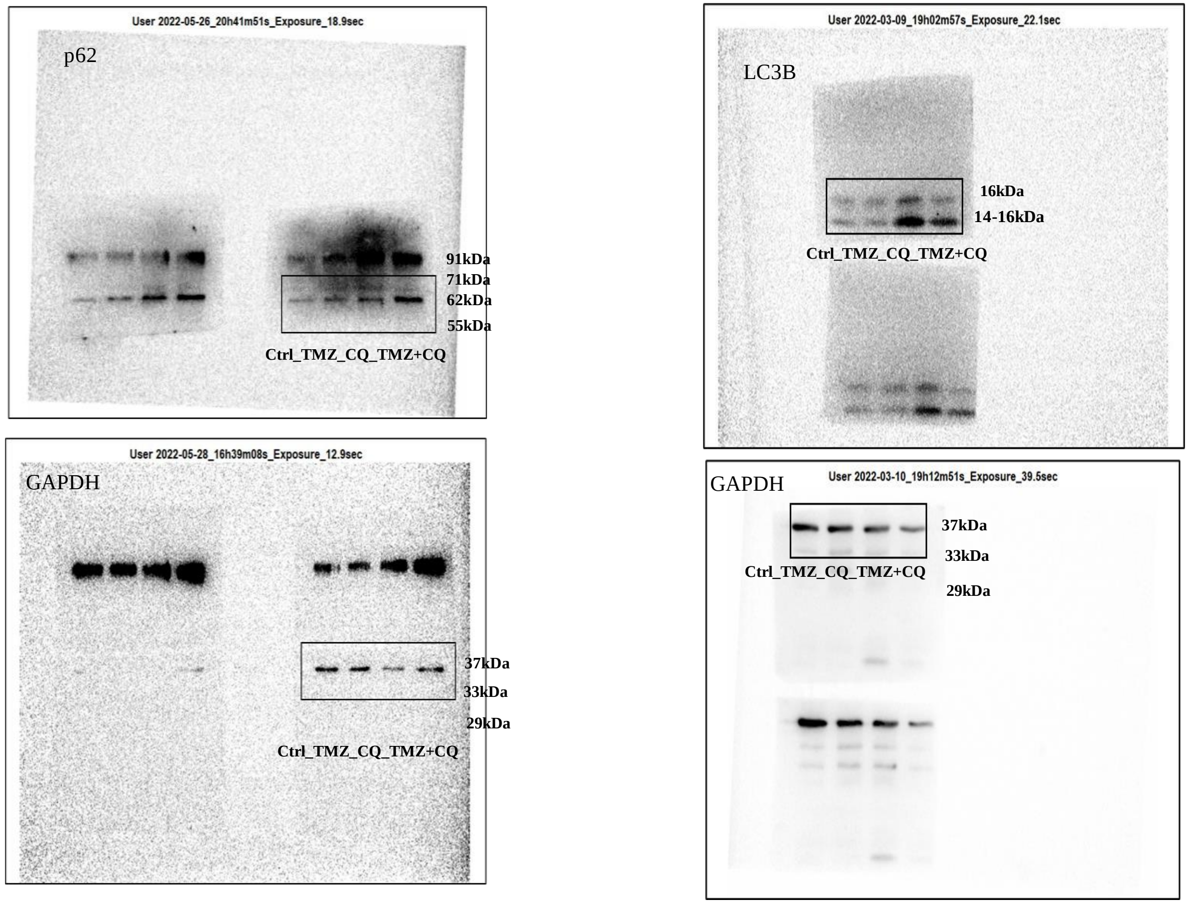

Figure 5.a.ii

Cell Line: U37
Treatment: TMZ and CQ
Timepoints:48 hours
Experiment: Western Blotting of p62 and LC3B
Software Used: Image Lab and Image J

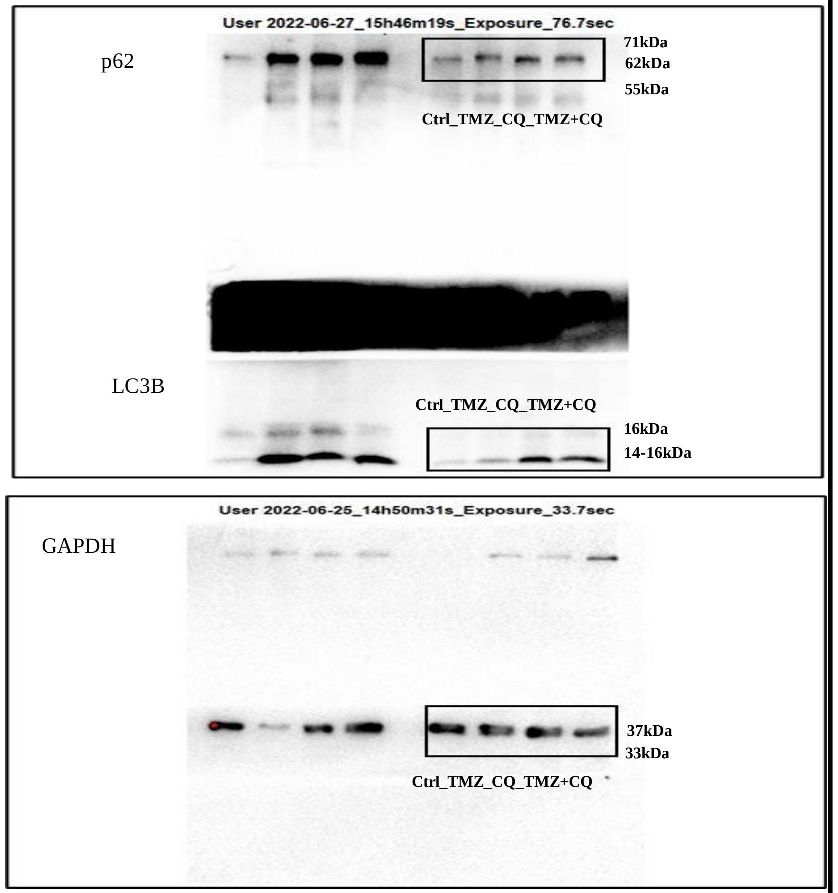

Cell Line: U87
Treatment: Doxy and Gem
Timepoints:48 hours
Experiment: Western Blotting of LAMP2A Software Used: Image Lab and Image J

Figure. 5f.i

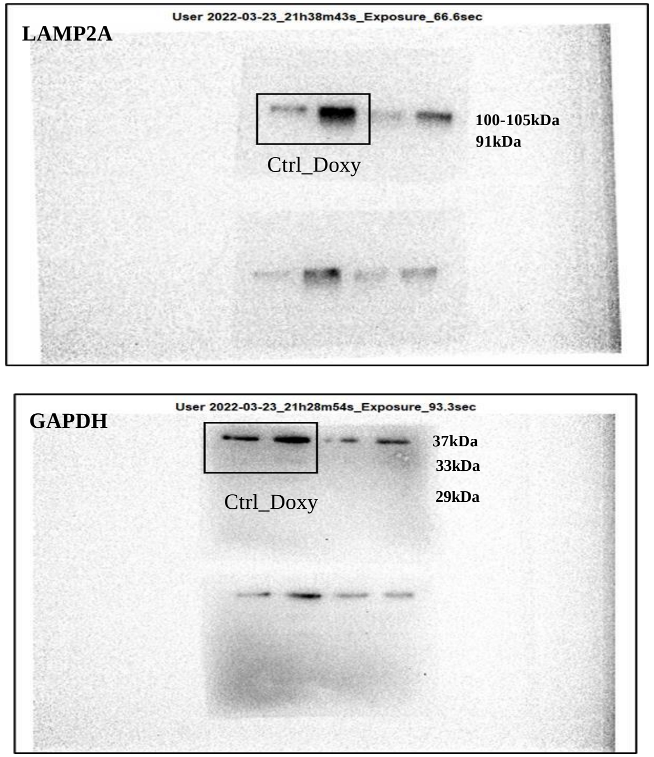

Figure. 5f.ii

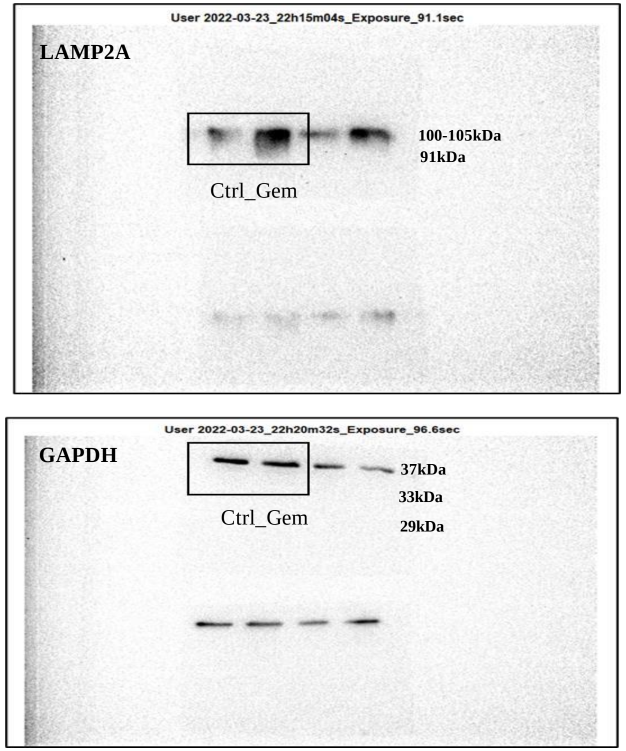

Figure. 5f.

Cell Line: U87 Treatment: Doxy and Gem
Timepoints: 5 days (Spheroids culture) Experiment:
Western Blotting of LAMP2A Software Used: Image Lab
and Image J

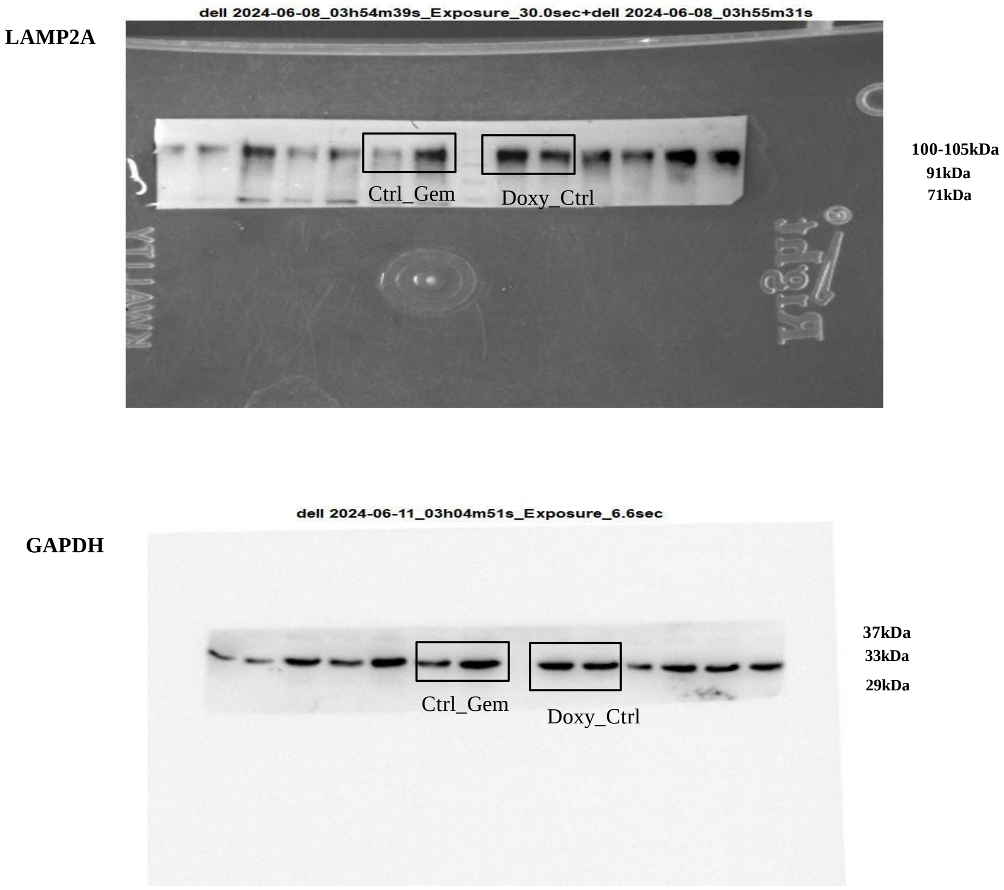

Figure. 5g.

Cell Line: U87 Treatment: Doxy, Gem, TMZ Timepoints:48 hours
Experiment: Western Blotting of p62 and LC3B Software Used: Image Lab
and Image J

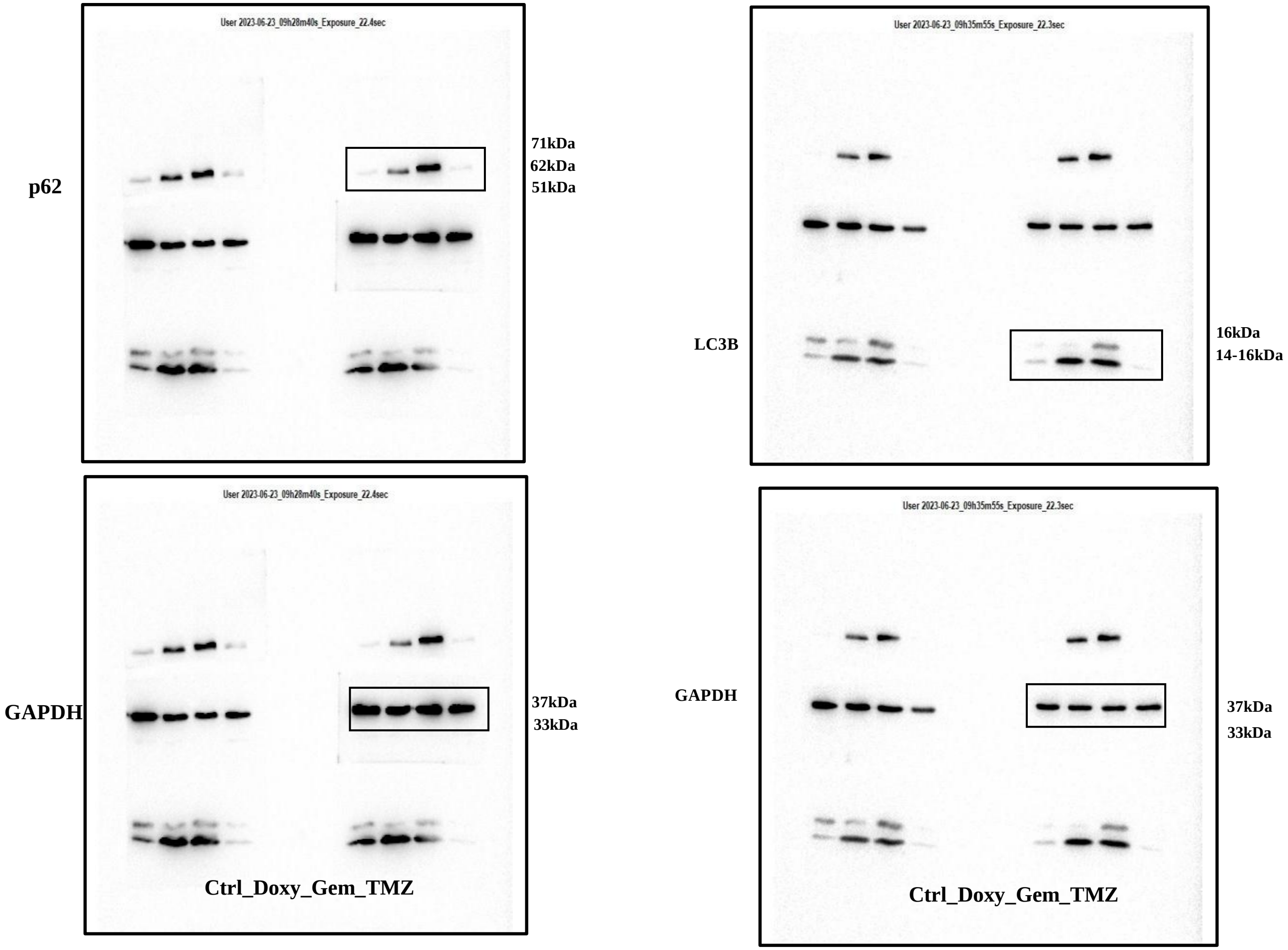

Figure. 5h.

Cell Line: U87 Treatment: Doxy and Gem Timepoints: 5 days (Spheroids culture) Experiment: Western Blotting of p62 and LC3 Software Used: Image Lab and Image J

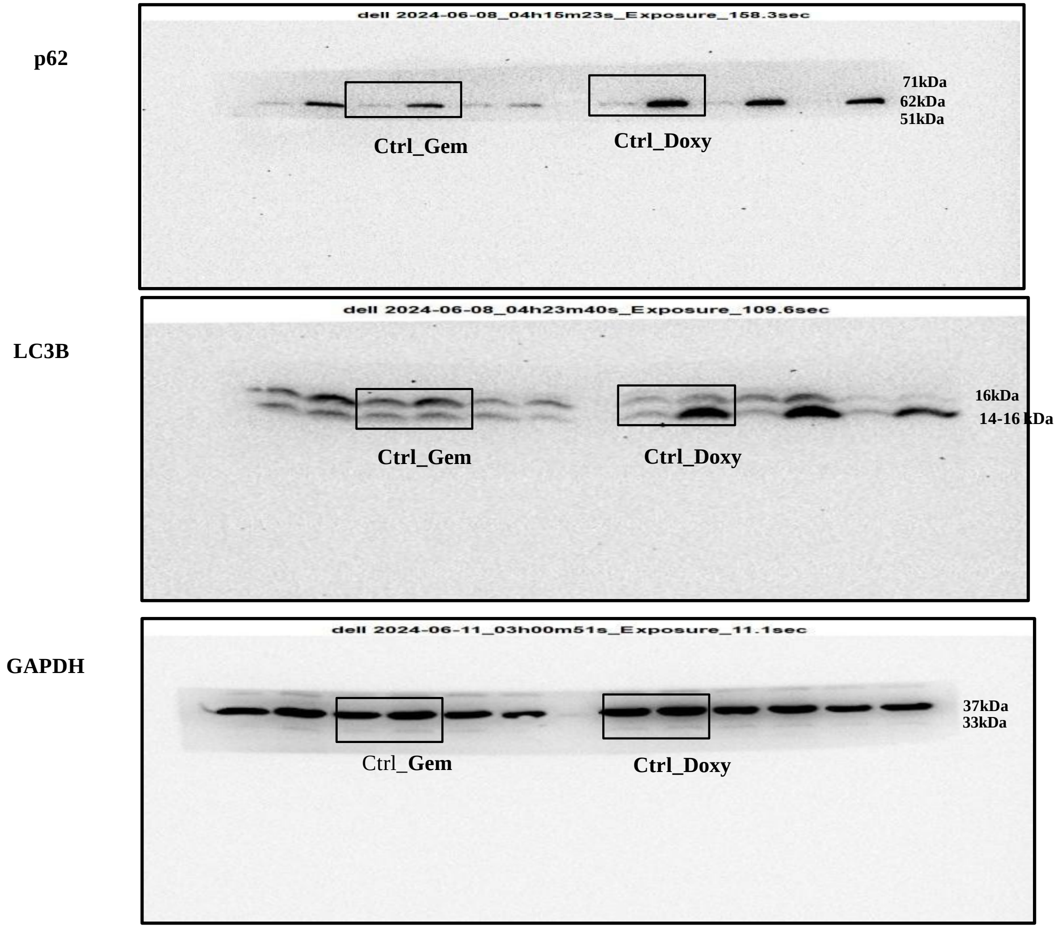

Figure. 7c.i, c.ii, c.iii, and c.iv

Cell Line: U87 **Treatment: Doxylamine, Gemfibrozil and NAC**
Timepoints: 48 hours
Experiment: Immunoblotting of p62 and LC3B Software Used: Image J and Image Lab

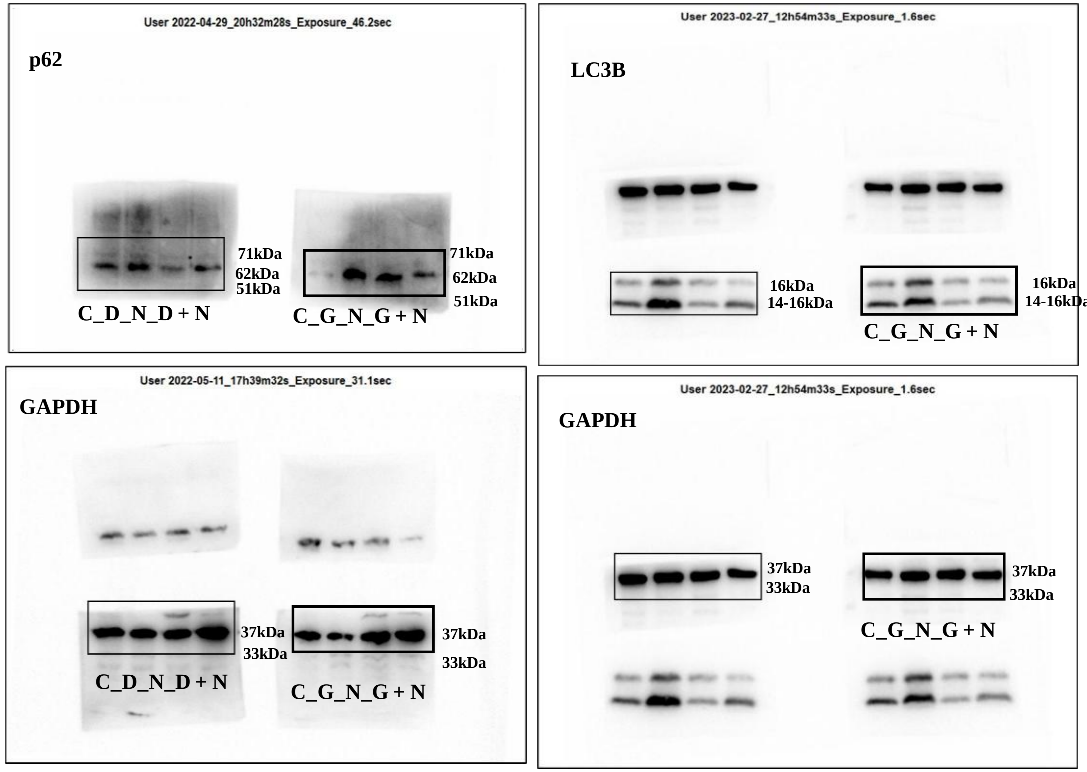

Supplementary Figure 1f.

Cell Line: U373 Treatment: Doxylamine, Gemfibrozil, VPA, Diprophylline and TMZ Timepoints:48 hours
Experiment: Western Blotting of PCNA
Software Used: Image Lab and Image J

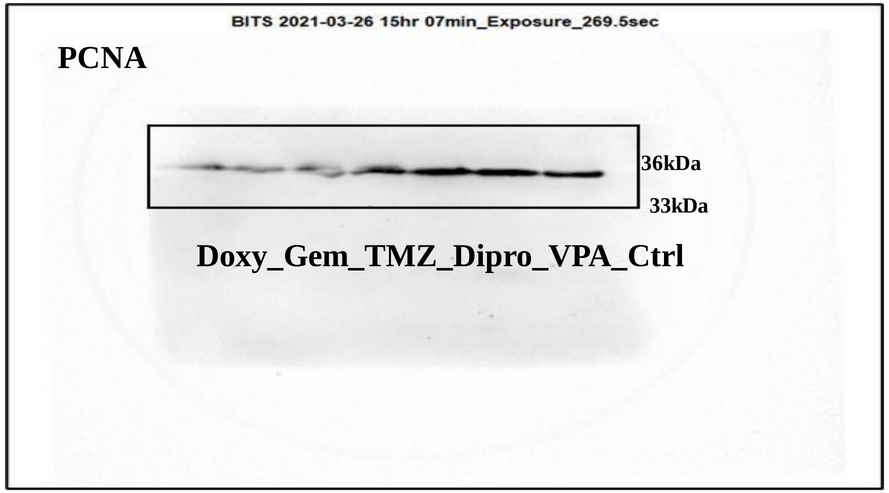

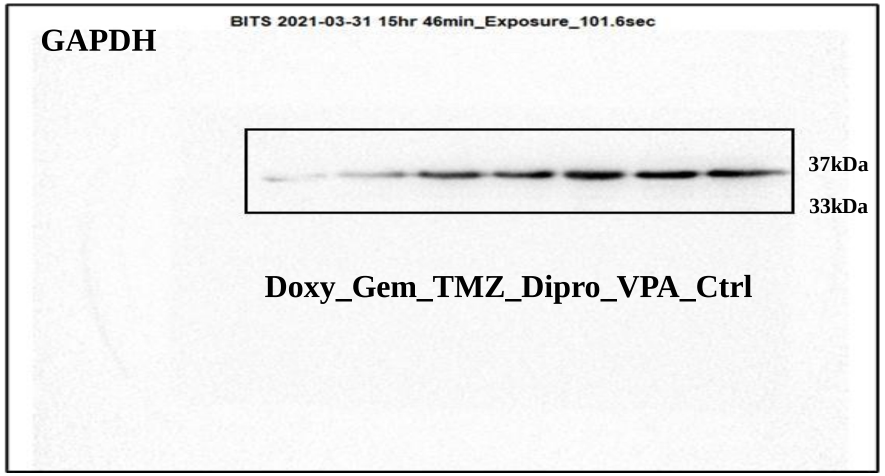

Supplementary Figure 1g.

Cell Line: U373 Treatment: Doxylamine, Gemfibrozil, and TMZ
Timepoints:48 hours
Experiment: Western Blotting of cCASPASE3
Software Used: Image Lab and Image J

cCASPASE3

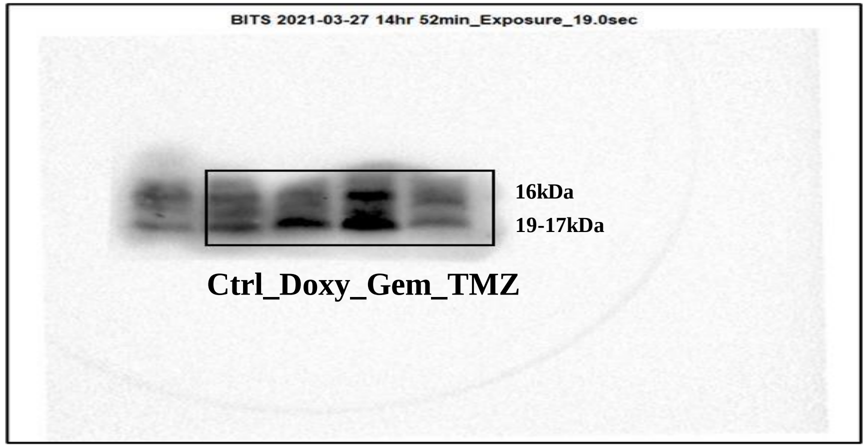

GAPDH

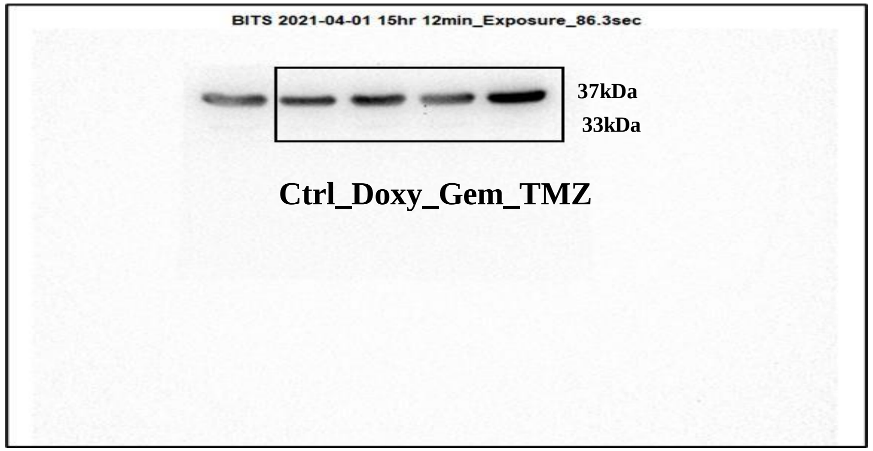

Supplement: Supplementary file 3 — Supplementary Material 3 [file 41598_2025_5054_MOESM3_ESM.pdf]
