# Supplementary material for: Repurposing of CNS accumulating drugs Gemfibrozil and Doxylamine for enhanced sensitization of glioblastoma cells through modulation of autophagy
Source: Sci Rep. 2025 Jul 1;15:20560. doi: 10.1038/s41598-025-05054-5 (PMC12219411; doi:10.1038/s41598-025-05054-5)
Supplement: Supplementary file 4 — Supplementary Material 4 [file 41598_2025_5054_MOESM4_ESM.docx]

**Supplementary Figure Legends:**

**Supplementary Figure 1: (a)** Crystal Violet images staining the viable cells post 24, 48, and 72 hours of VPA and Dipro treatment in the U87 cell line, **(b)** MTT assay showing the dose and time kinetics of Doxy, Gem, Dipro and VPA in U373 cells, **(c,d)** Phase contrast and Crystal violet images of U373 cells post-drug treatment, **(e.i.)** AnnexinV/PI assay confirming the toxicity of the drugs after 48 hours of incubation **(e.ii.)** Bar graph plotted of the AnnexinV/PI data **(f)** Western blot expression of proliferation marker PCNA confirmed Doxy and Gem inhibit U373 cell proliferation, **(g)** Cleaved Caspase 3 expression increased post-Doxy and Gem treatment validated the drug activity, **(h)** Crystal Violet images of viable cells post Co-treatment of Doxy/Gem and TMZ in U373 cells confirmed the synergy between the drugs. The scale bar represents 10 µm. * is used to represent the significant p-values.

**Supplementary Figure 2:** (a) Bar graph representing the size of the Spheroids after 0, 1, 3, 5, and 7 hours of Doxylamine, Gemfibrozil, VPA, and TMZ treatment, (b) Bar graph representing the size of Spheroids after combination treatment of Doxy/or Gem and TMZ.

**Supplementary Figure 3:** A flowchart illustrating the identification of hub genes and corresponding pathway analysis based on patient-derived data from the GEO datasets GSE2223 and GSE4290.
